# 3-methylcrotonyl Coenzyme A (CoA) carboxylase complex is involved in the *Xanthomonas citri* subsp. *citri* lifestyle during citrus infection

**DOI:** 10.1371/journal.pone.0198414

**Published:** 2018-06-07

**Authors:** Mauro Tomassetti, Betiana S. Garavaglia, Cecilia V. Vranych, Natalia Gottig, Jorgelina Ottado, Hugo Gramajo, Lautaro Diacovich

**Affiliations:** Instituto de Biología Molecular y Celular de Rosario (IBR-CONICET), Facultad de Ciencias Bioquímicas y Farmacéuticas, Universidad Nacional de Rosario, Rosario, Argentina; Fujian Agriculture and Forestry University, CHINA

## Abstract

Citrus canker is a disease caused by the phytopathogen *Xanthomonas citri* subsp. *citri* (Xcc), bacterium which is unable to survive out of the host for extended periods of time. Once established inside the plant, the pathogen must compete for resources and evade the defenses of the host cell. However, a number of aspects of Xcc metabolic and nutritional state, during the epiphytic stage and at different phases of infection, are poorly characterized. The 3-methylcrotonyl-CoA carboxylase complex (MCC) is an essential enzyme for the catabolism of the branched-chain amino acid leucine, which prevents the accumulation of toxic intermediaries, facilitates the generation of branched chain fatty acids and/or provides energy to the cell. The MCC complexes belong to a group of acyl-CoA carboxylases (ACCase) enzymes dependent of biotin. In this work, we have identified two ORFs (XAC0263 and XAC0264) encoding for the α and β subunits of an acyl-CoA carboxylase complex from *Xanthomonas* and demonstrated that this enzyme has MCC activity both *in vitro* and *in vivo*. We also found that this MCC complex is conserved in a group of pathogenic gram negative bacteria. The generation and analysis of an Xcc mutant strain deficient in MCC showed less canker lesions in the interaction with the host plant, suggesting that the expression of these proteins is necessary for Xcc fitness during infection.

## Introduction

**Fig 1:**
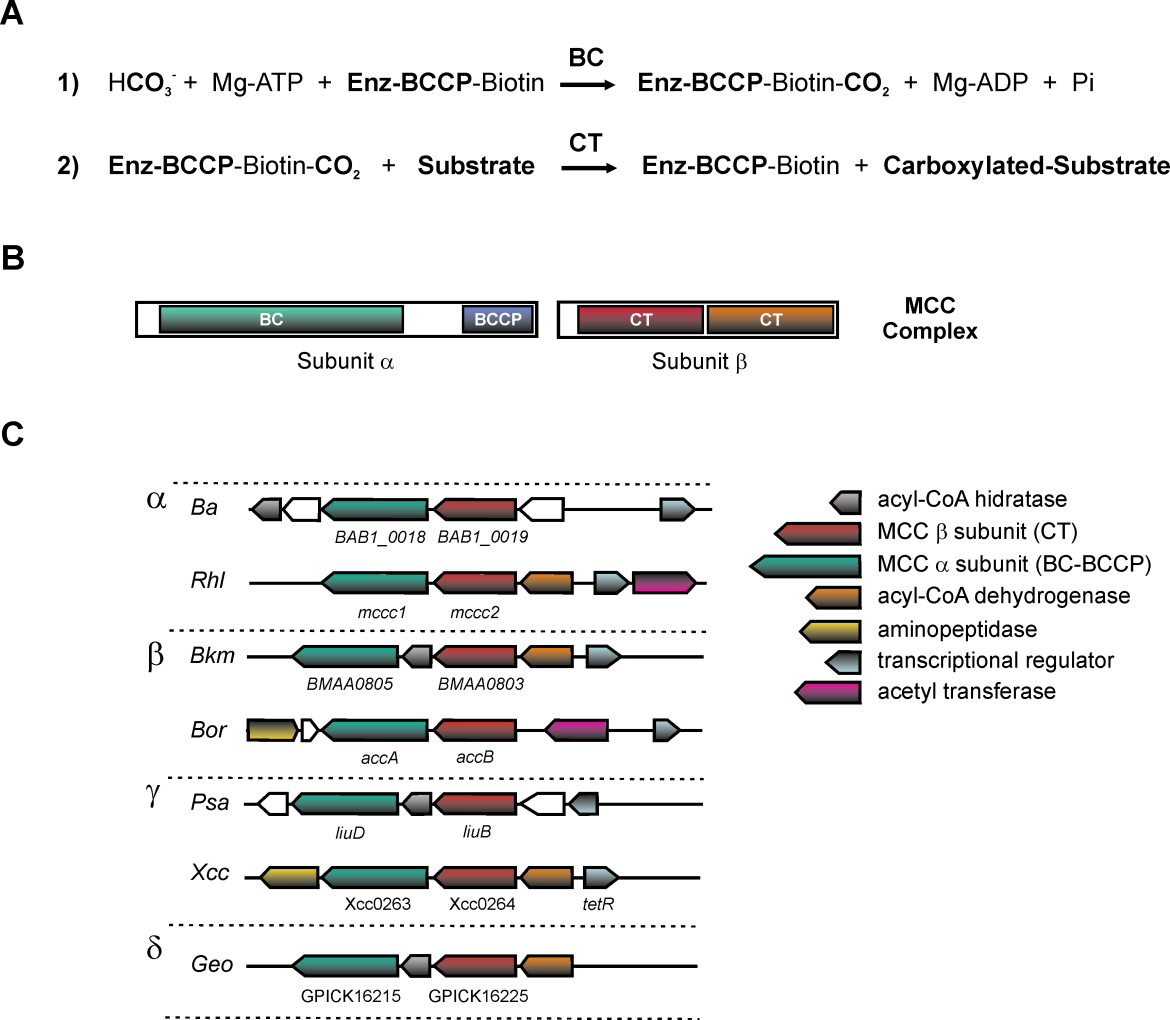
Schematic diagram of domains, activity, subunits, and genetic organization of the biotin-dependent MCC from Xcc. (A) The biochemical activity of ACCases takes place in two sequential reactions. In the first reaction, a biotin carboxylase (BC) component catalyzes the carboxylation of the cofactor biotin in Mg^2+^ and ATP dependent step. Biotin is covalently linked to a conserved residue of lysine in the biotin carboxyl carrier protein (BCCP) component. In the second step, the carboxyltransferase (CT) component catalyzes the carboxyl transfer from carboxybiotin to the acyl-CoA acceptor. (B) MCC enzymatic complex is composed of three different components, the BC component, the BCCP and CT. In bacteria, MCC are mainly made up of two main subunits: α subunit, which contains the BC and BCCP components and β subunit, which has the CT activity. (C) Synteny present in the cluster of genes encoding for MCC putative subunits, and other enzymes for leucine catabolism, including an acyl-CoA hydratase, acyl-CoA dehydrogenase, aminopeptidase and a transcriptional regulator, in relevant species of α, β, γ, δ proteobacterias. *Ba*, *Brucella abortus*; *Rhl*, *Rhizobium leguminosarum bv*. *viciae; Bkm*, *Bulkholderia mallei*; *Bor*, *Bordetella pertusis*; *Psa*, *Pseudomonas aeruginosa*; Xcc, *Xanthomonas citri subsp*. citri; *Geo*, *Geobacter picjeringii*.

In most of the organisms, including bacteria, archaea, fungi, algae, plants, and animals, the enzymatic complexes of biotin-dependent carboxylases catalyze fundamental metabolic reactions. These reactions are involved in the metabolism of fatty acids, carbohydrates, and amino acids, as well as in polyketide biosynthesis, urea utilization [1–4]. Biotin-dependent carboxylases contain three different components: the biotin carboxylase (BC), the biotin carboxyl carrier protein (BCCP) and the carboxyltransferase (CT). These components catalyze two separate hemi-reactions [5,6] (Fig 1A).

The BC component catalyzes the first half-reaction, which involves the phosphorylation of bicarbonate by ATP to form a carboxyphosphate intermediate, followed by transfer of the carboxyl group to the biotin to form carboxybiotin [7–10]. In the second reaction, catalyzed by the CT component, the carboxyl group is transferred from the carboxy-biotin to the substrate to form a carboxylated product [11–15]. A thumb-like loop region on BCCP, enables the lysine-biotin conjugate to move alternatively between the BC and CT domains, in order to transport the carboxyl group [16]. This family of enzymes uses coenzyme A (CoA) esters of mostly short-chain organic acids as their substrates. The carboxylation occurs either in the α carbon of saturated acids, for example acetyl- or propionyl-CoA, or the γ carbon of the α-β unsaturated acid, such as 3-methylcrotonyl-CoA or geranyl-CoA. In general, these enzymes are referred to as acyl-CoA carboxylases (ACCases or YCC), where the substrate specificity is defined by the CT component [17–20]. ACCases were proposed as interesting targets for drug discovery against microbial infections, obesity, cancer, and type 2 diabetes [15].

In most of the organisms, the carboxylation of 3-methylcrotonyl-CoA to generate 3-methylglutaconyl-CoA, catalyzed by the enzyme 3-methylcrotonyl-CoA carboxylase (MCC), is an essential step for the catabolism of leucine and isovalerate [15,21,22–25]. The final products of this pathway are acetyl- and acetoacetyl-CoA, which can be reintroduced into different metabolic pathways in situations of nutritional stress or amino acid excess. Deficiency in the MCC activity of is linked to serious diseases in humans [22–25]. The role of these enzymes in Gram-negative bacteria was only studied in *Pseudomonas*, where MCC is involved in the metabolism of acyclic terpenoids [26–31].

Acetyl-CoA carboxylase (ACC) and propionyl-CoA carboxylase (PCC) enzyme complexes from actinomycetes and MCC from *Pseudomonas* share significant homology at the amino acids level and also a conserved quaternary structure [15,32–34]. All of them consist of two subunits, a larger one (α-chain) containing the BC and BCCP domains with the ability to carboxylate its covalently bound biotin group, and a smaller subunit (β-chain) bearing the CT activity (Fig 1B). MCC and PCC holoenzymes are 750-kDa α_6_β_6_ dodecamers. Crystal structures of MCC holoenzyme from *P*. *aeruginosa* and PCC enzymes revealed a conserved β_6_ hexamer core with three α subunits at each end (α_3_β_6_α_3_) [34].

The presence and the role of ACCases in phytopathogenic bacteria had not yet been addressed. The bacterial genus *Xanthomonas* comprises a number of Gram-negative plant pathogenic bacteria that cause a variety of severe plant diseases [35]. The genus *Xanthomonas* has become an important model organism for studying plant–microbe interaction and for understanding bacterial pathogenicity and virulence mechanisms [36]. Most of the current studies related to the interactions between *Xanthomonas* and host cells have focused on microbial virulence factors [37,38]. However, there are very few reports about the characterization of the metabolism of plant pathogens during infection [36,39–42].

*Xanthomonas citri* subsp. *citri* (Xcc) is the phytopathogen responsible for citrus canker, a severe disease that affects all commercial citrus cultivars causing serious economic losses [43]. Xcc enters the host tissue and colonizes the apoplast of leaves, stems and fruits. Once established inside the plant, the pathogen must compete for resources and evade the defenses of the host cell. In this work we have identified two ORFs encoding for α (AccC) and β (AccD) subunits of an acyl-CoA carboxylase complex from Xcc and demonstrated that this enzyme has MCC activity both *in vitro* and *in vivo*. An Xcc mutant deficient in MCC activity produced less canker lesions than the wild type Xcc in the interaction with the host plant, suggesting that this complex is relevant for Xcc lifestyle into the host. Our results show for the first time a role for these enzymes in the phytopathogen infection process.

## Results

### Predictive analysis of the putative MCC of *Xanthomonas*: Identification of α and β subunits

In order to identify genes encoding for α and β subunits of MCC orthologous complexes in Gram-negative bacteria, we performed a bioinformatic search using the amino acid sequences of the MCC subunits previously characterized in human and in *Pseudomonas aeruginosa* [15,29,31,34]. Using the blastP program, we found proteins with a high sequence identity (>46%) in different pathogenic bacteria, as *Xanthomonas*, *Bordetella*, *Klebsiella*, *Burkholderia* (S1 Table) and *Mycobacterium* [44,45]. Particularly Xcc has two ORFs, named XAC0263 and XAC0264, encoding for the putative α and β subunits of an ACCase complex in this bacterium, respectively. The α subunit displays four highly conserved domains present in the α subunits of several ACCases already identified [34]: (i) the ATP binding site (GGGGKGM, 175–181), (ii) the CO_2_ fixation domain (RDCS, Cys242), (iii) the catalytic site of the biotin-dependent carboxylase family (EMNTR), and (iv) the biotin-carboxyl carrier domain (EAMKM, E635-M639) (S1 Fig). On the other hand, the β subunit presents a high degree of conservation with the amino acidic residues that interact with 3-methylcrotonyl-CoA in the MCC from *Pseudomonas*. Ala46, Ala50, Lys113, Thr116, Leu455, and Val458 could interact with the S-CoA cofactor, while Ala148, Phe163, and Gly191 could form the pocket to support one of the γ carbons of the substrate (S2 Fig). Also Phe380, Gly421, Ala148 and Gly191 are highly conserved residues and could form the two oxyanions necessary for the catalysis. We also analyzed the genetic organization of the genes encoding for α and β subunits of these putative ACCase complexes in the different proteobacteria. In Xcc, the genes XAC0263 and XAC0264, also annotated as *accC* and *accD*, respectively, are clustered in a locus together with genes encoding for an acyl-CoA dehydrogenase (XAC0265), an aminopeptidase (XAC0262) and a putative response regulator (XAC0266). This genetic organization is similar to the one found for the *liuRABCDE* cluster from *P*. *aeruginosa*, which has been proposed to be involved in leucine and isovalerate catabolism [28]. This locus shows a partial synteny with the orthologous genes from other proteobacteria (Fig 1C).

Promoter analysis of the upstream region of *accC* and *accD* was performed with MEME/MAST software to search for signatures [46]. This analysis revealed that both *accC* and *accD* contain a putative-imperfect PIP box (TTCGC-N15-TTCGC). PIP boxes are plant inducible promoter elements (PIP) that are recognized by the product of the *hrpX* gene, which regulates the expression of genes involved in pathogenicity [47].

### *In vitro* reconstitution of the MCC-Xcc complex from *E*. *coli* extracts

The *in vitro* reconstitution of heterologous ACCase complexes using *E*. *coli* cell extracts is a well-established method to measure their enzymatic activity [20]. Indeed, to asses if the predicted ACCase complex from Xcc is functional we performed *in vitro* reconstitution experiments using crude *E*. *coli* extracts expressing Xcc proteins AccC and AccD.

**Fig 2:**
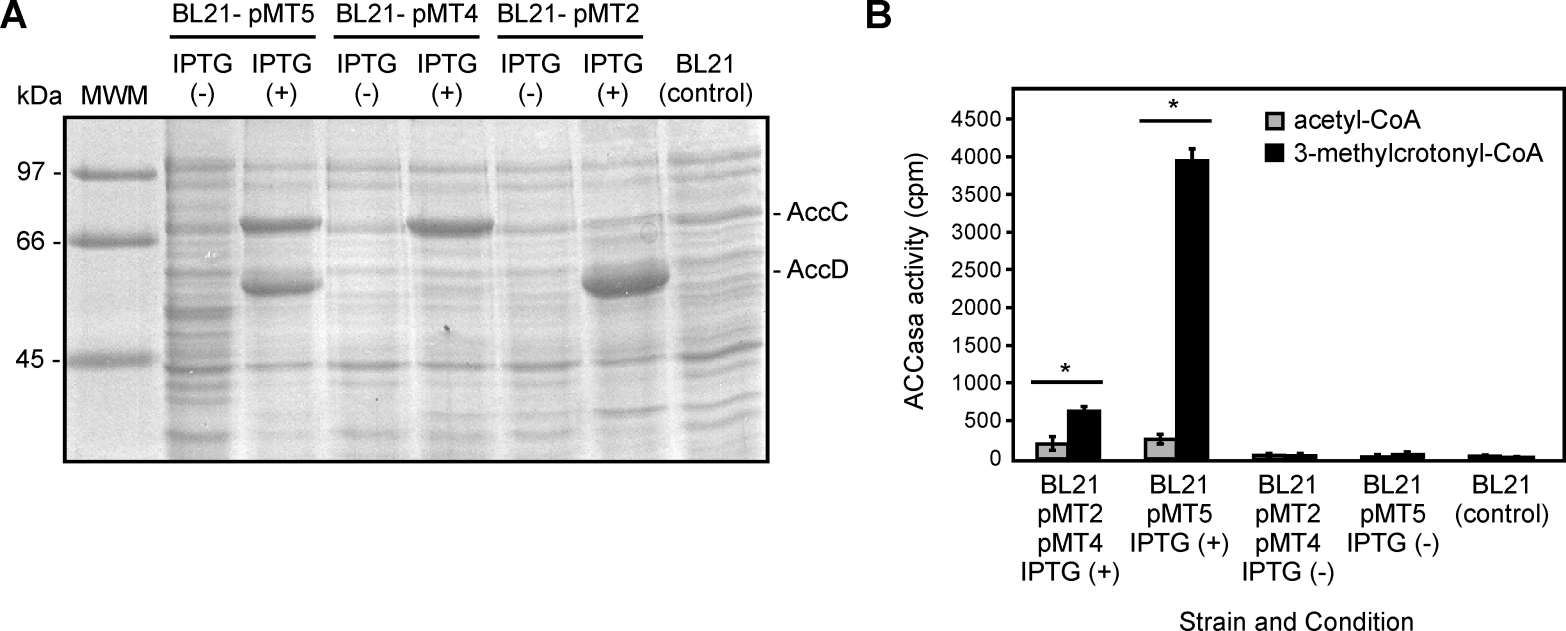
Heterologous expression of α y β subunits from the Xcc MCC complex in *E*. *coli* and ACCase activity measures. (A) SDS-PAGE with total extracts from BL21 *E*. *coli* strains carrying the plasmids pMT5, pMT4 and pMT2, expressing AccC and AccC proteins, in presence or absence of the inductor IPTG. A BL21 strain without plasmid was used as a control. MWM, molecular weight marker. (B) ACC and MCC activities were measured after mixing equal amounts of proteins from cell extracts from each of the strains indicated. Results are the means of three determinations. When ACCase activities were measured in individual cell extracts from BL21-pMT2 or BL21-pMT4, the amount of ^14^C fixed into acid-stable products was not significantly higher than background levels obtained with the control strain BL21 without plasmid (40 cpm). Values are means ± SD of 3 independent experiments. Unpaired t-test was used to determine whether two values were significantly different. *P*-values: *, *P* < 0.05.

Overexpression of His-AccC and His-AccD, or the individual proteins was performed in BL21 (DE3) cells containing the plasmids pMT5, pMT4, or pMT2, respectively. SDS-PAGE of crude extracts prepared from IPTG-induced cultures of these strains, revealed overexpression of 72 and 57 kDa proteins, corresponding to the predicted size of His-AccC and His-AccD, respectively (Fig 2A). *In vitro* reconstitution of ACCase activity was assayed by mixing crude extracts prepared from the IPTG-induced culture of the strains containing the individual subunits, or cell extracts from the strain overexpressing both subunits, His-AccC and His-AccD. After incubation for 15 min at 25°C, the mixture was assayed for ACC and MCC activities. As shown in Fig 2B, MCC activity was readily detected, suggesting that AccC and AccD are the BC and the CT components of an Xcc MCC complex. However, very low levels of ACC activity were detected when acetyl-CoA was used as a substrate. Interestingly, when the complex was reconstituted from the BL21 strains harboring the plasmid pMT5, which expresses both subunits, the MCC activity recovered was considerably higher than those obtained from the mix of the strains expressing AccC and AccD separately. This result suggest that the MCC complex could be stabilized when the two protein are coexpressed, allowing a maximal activity.

### Reconstitution of the MCC complex from its purified subunits: Stability and stoichiometric analysis

**Fig 3:**
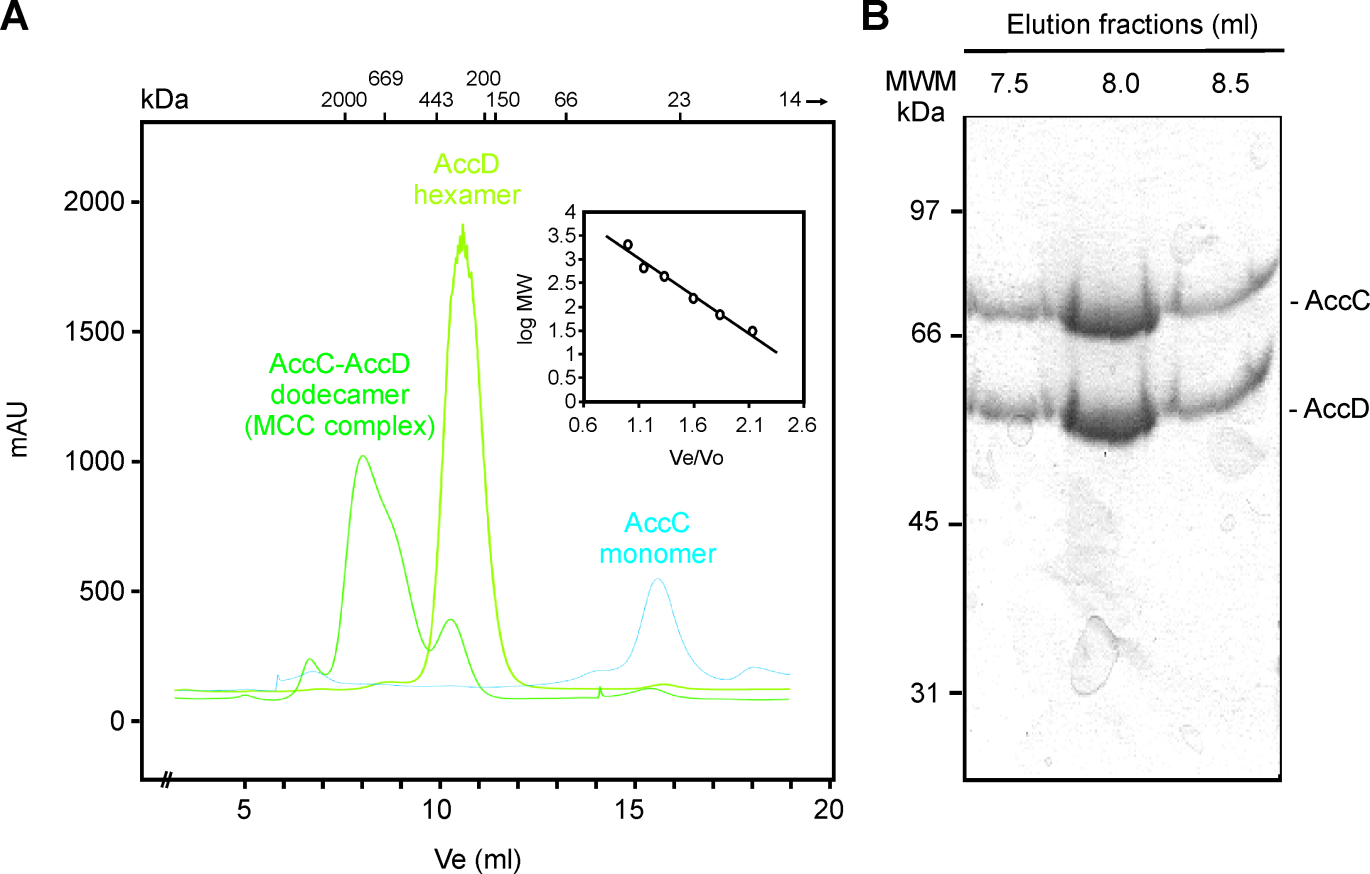
Study of MCC complex by size exclusion chromatography. (A) The oligomeric state of the individual subunits, AccC and AccD, were analyzed by size exclusion chromatography. The stoichiometry of the α-β MCC complex was also determined by running in the column a preincubated mixture of both proteins. The protein profiles were followed by measuring absorbance at 215 nm (in milli-absorbance units [mAU]). The molecular masses (kDa) corresponding to the protein standards used for calibration curve are indicated at the top of the figure. The calibration curve is shown in the inset. (B) SDS-PAGE analysis of the elution fractions corresponding to the α-β MCC complex. MWM, standard molecular weight markers.

To confirm the hypothesis that the AccC and AccD proteins are the α and β subunits of the Xcc MCC complex, we expressed and purified the two putative components of the complex, as N terminal His-tagged proteins from *E*. *coli*. The purification of His-AccD was successful, recovering the protein in a final concentration of 2.5 mg ml^-1^ (S3 Fig, panel A). The purification of the α-subunit, His-AccC, resulted in lower levels of protein, with a final concentration of 0.34 mg ml^-1^ (S3 Fig, panel B), and presenting high quantities of AccC protein in the precipitated fraction. Since α and β subunits would form a stable complex the yield of both subunits was improved by co-expressing them in *E*. *coli* cells transformed with the pMT5 vector. A complex formed by His-AccD and His-AccC was successfully purified at a final concentration of 9.4 mg ml^-1^ (12.5 μM), (Fig 3 and S3 Fig, panel C). This experiment suggests that the co-expression of AccC and AccD proteins improves the levels and the stability of both subunits, increasing the α-subunit solubility in *E*. *coli* extracts. Furthermore, we also verified by Western-Blot analysis that the His-AccC protein obtained was biotinylated (S3 Fig, panel D).

The oligomeric state of the individual subunits AccC (α) and AccD (β) and the molar ratio of these proteins in the MCC complex were determined by using a size exclusion chromatography. His-AccC runs as a monomer in solution, while the elution profile of His-AccD indicates that this protein is a hexamer (Fig 3A). When the two subunits were mixed a new chromatographic peak was identified, and the calculated molecular mass of this peak corresponded to a heterododecamer probably containing by six subunits of His-AccC and six subunit of His-AccD. The analysis of the elution fractions by a SDS-PAGE indicates that both subunits, AccC and AccD, are present in 1:1 stoichiometry in those fractions corresponding to the complex. Previous studies on the *P*. *aeruginosa* MCC [34] and others ACCase complexes [17–20] have also indicated a α_6_/β_6_ structure.

### Kinetic parameters of the MCC complex

**Fig 4:**
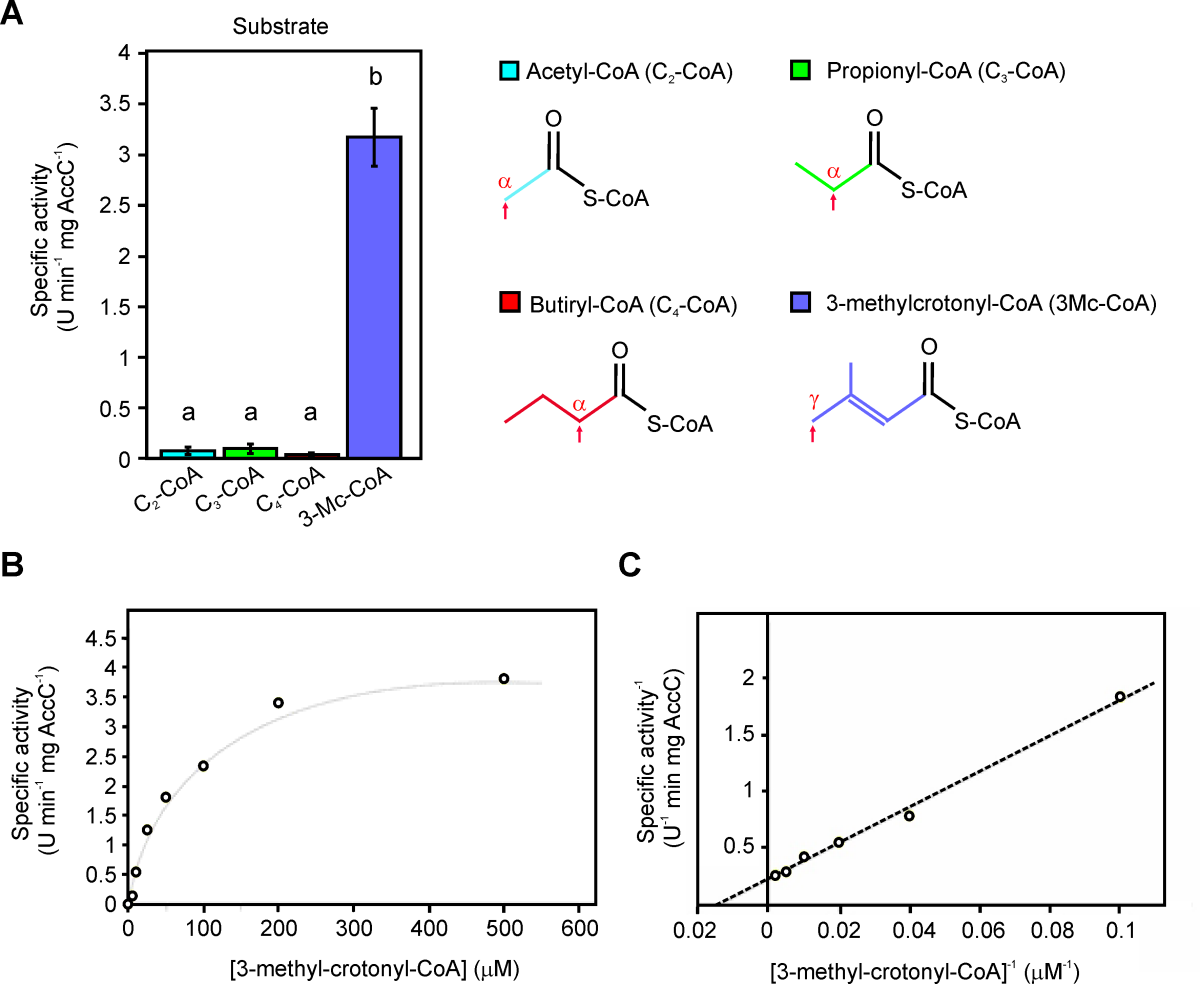
*In vitro* reconstitution of the MCC activity from the purified components. (A) The co-purified subunits AccC and AccD were incubated with different acyl-CoA substrates at a concentration of 0.5 M, including acetyl- (blue), propionyl- (green), butiryl- (red) and 3-methyl-crotonyl-CoA (violet). The chemical structure of the substrates are shown, the position (α or γ) of the carboxylated carbon in each case is indicated by an arrow. Values are means ± SD of 3 independent experiments. One-way ANOVA and Tukey post-tests were used to determine whether the values were significantly different. Different letters (a and b) indicate statistically significant differences between groups (mean ± SE). *P*-values: a vs. b, *P* < 0.05. (B) The kinetic characterization of the MCC complex (0.5 μM) was performed using the PK-LDH coupled assay. Curve of specific activity in function of substrate concentration is shown. The data presented a best fit to a hyperbolic curve. Results are the means of three independent experiments. (C) Double reciprocal plot used for the estimation of the kinetic parameters *K*_*m*_ and *V*_*max*_.

By using the fraction containing the purified dodecameric complex, four different substrates were assayed in order to determine the substrate specificity of this ACCase complex. Very low activity levels were detected when mixtures containing 0.05 μg ml^-1^ of AccC-AccD, at a 1:1 molar ratio, were assayed with saturating concentration (0.5 mM) of acetyl-, propionyl-, or butyryl-CoA. However, the enzyme was significantly more active when 3-methylcrotonyl-CoA was used as a substrate (Fig 4A), confirming that these proteins constitute a functional MCC complex. The kinetics parameters of the reconstituted enzyme complex were determined resulting in values of *K*_*m*_, *V*_max_, specificity constant, and catalytic efficiency (*K*_*cat*_*/K*_*m*_) of 71.4 μM, 4.5 U min^-1^ mg AccC^-1^, 62.7, and 5887 M^-1^ s^-1^, respectively (Fig 4B and 4C). These values are comparable to those reported for others ACCase complexes characterized [48–50].

### Measurement of MCC activity from Xcc extracts

**Fig 5:**
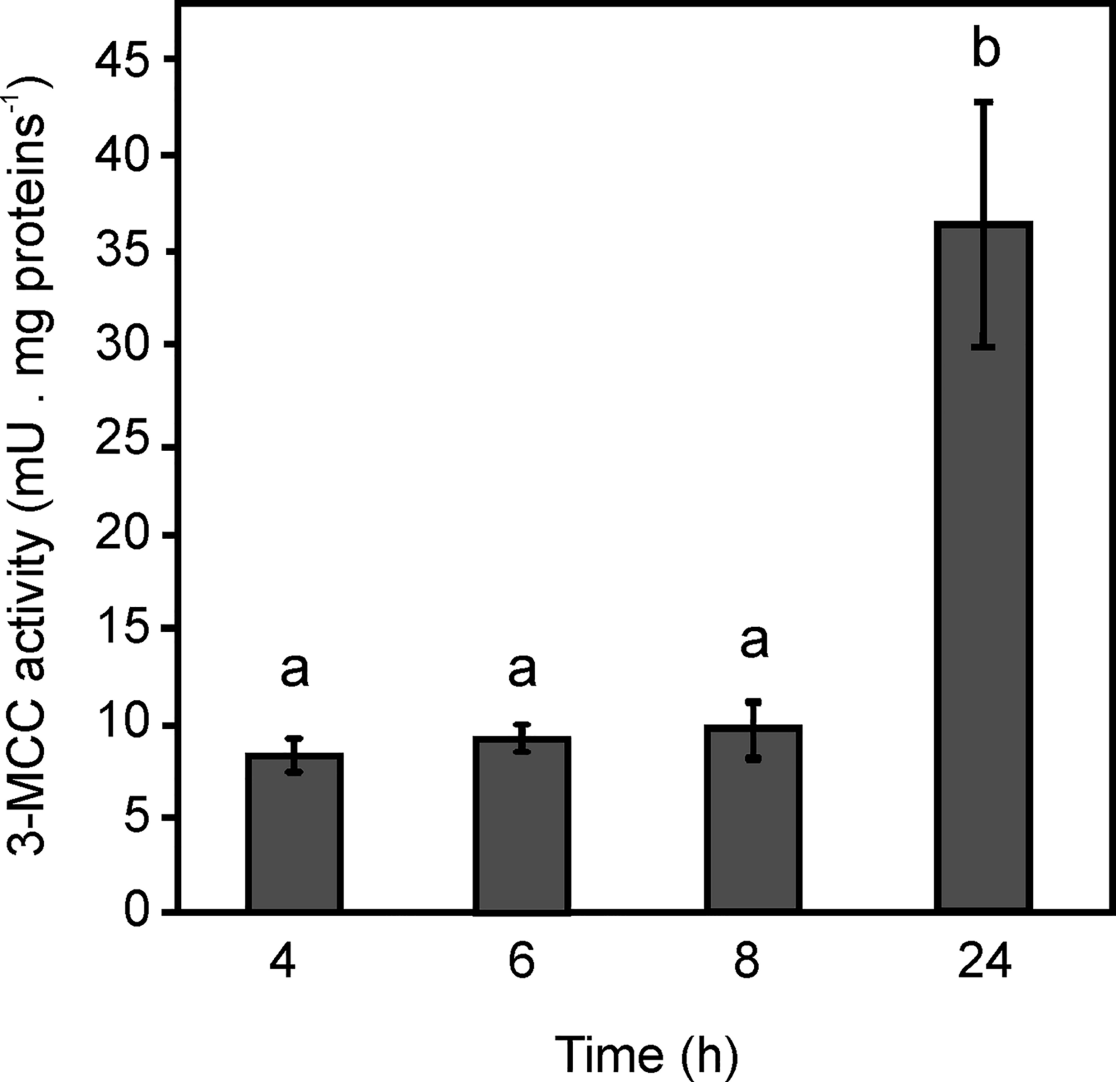
MCC activity reconstituted from Xcc cell-free extracts. MCC activity was measured, using the radioactive assay, from cell extracts of Xcc grown in NB medium at different time points. Results are the means of three independent experiments ± standard deviations (n = 3). One-way ANOVA and Tukey post-tests were used to determine whether the values were significantly different. Different letters (a and b) indicate statistically significant differences between groups (mean ± SE). *P*-values: a vs. b, *P* < 0.05.

In order to study if the Xcc MCC complex is active when the bacterium is grown in axenic cultures, MCC activity was evaluated in cell-free extracts obtained from Xcc grown in NB medium at different time points of the exponential and stationary growth phases (S4 Fig). Similar levels of MCC activity were measured at 4, 6 and 8 hours of exponential growth (Fig 5). However, at the stationary phase (24 hours), a 2.1 fold increase of the enzyme activity was detected (Fig 5). Values were relativized to the control reaction in presence of 3-methylcrotonyl-CoA as a substrate and without cell extract.

### Expression profiles of *accC* and *accD* genes

**Fig 6:**
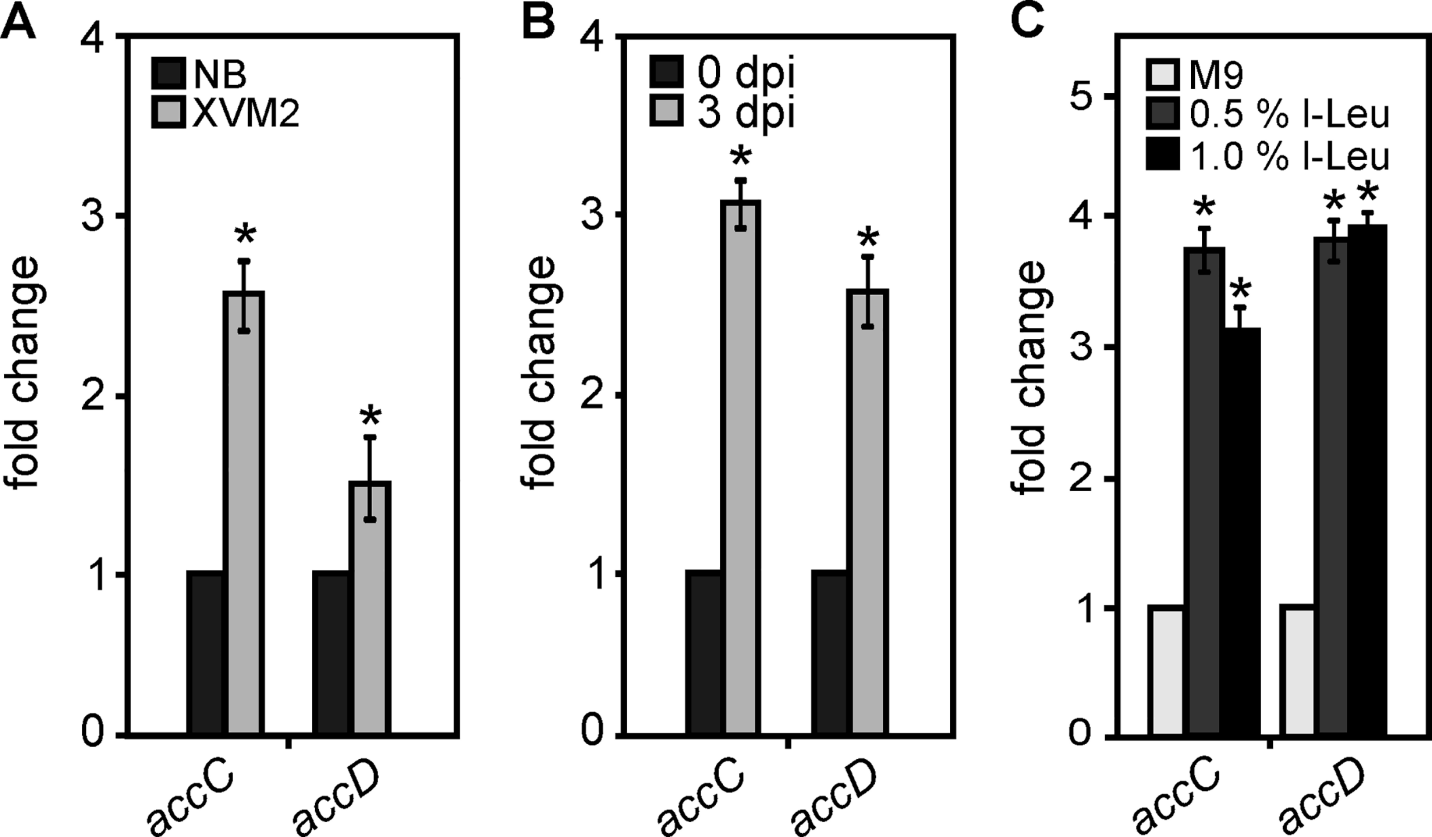
Analysis of *accC* and *accD* expression in different growth conditions and during infection. Xcc was cultivated in NB or XVM2 medium (A), recovered from inoculated orange leaves at 0 or 3 days post-infection (B), or cultivated in M9 medium supplemented with 0.5 and 1% (w/v) l-leucine (C). In each case, RNA was obtained from the bacteria and the expression of *accC* (XAC0263) and *accD* (XAC0264) was quantified by RT-PCR. The same RT-PCR conditions were utilized for the amplification of a fragment of 16S rRNA, employed as constitutive control. The graphics represent band intensity relative to the control. The experiments were repeated three times with similar results. In each case bars represent means of the three experiments and error bars represent standard deviation. Unpaired t-test was used to determine whether a value was significantly different from the control. *P*-values: *, *P* < 0.05.

Considering the results obtained in the *in vitro* MCC activity assays we proceeded to evaluate if the *accC* and *accD* genes were differentially expressed in different growth conditions. Therefore, we analyzed by RT-PCR the expression of both genes in rich (NB) and in the minimal medium (XVM2), which simulates conditions in the plant apoplastic space [51]. As observed in Fig 6A, the expression of *accC* and *accD* genes was 2.6 and 1.4 fold higher respectively, in XVM2 medium compared to their levels in rich medium (*p* < 0.05). Then the expression profiles of *accC* and *accD* were analyzed in the context of the plant infection. For this, leaves of *Citrus sinensis* were infected with Xcc cells, and RNA was purified from bacteria recovered from infected leaves, at cero and three days post inoculation (dpi). The levels of *accC* and *accD* mRNA were assayed by RT-PCR. As shown in Fig 6B, the expression of both genes was increased around two-fold at three dpi. Considering that the MCC complex could be involved in l-Leu metabolism in this bacterium, we analyzed if *accC* and *accD* genes were differentially expressed in presence of l-leucine in the medium. For this, Xcc was grown in M9 medium supplemented or not with 0.5 and 1% (w/v) l-Leu. RT-PCR reactions carried out on RNA purified from the three different growth conditions showed that both genes were induced in the presence of l-leu (Fig 6C).

### Construction and characterization of an Xcc *accD* mutant strain

**Fig 7:**
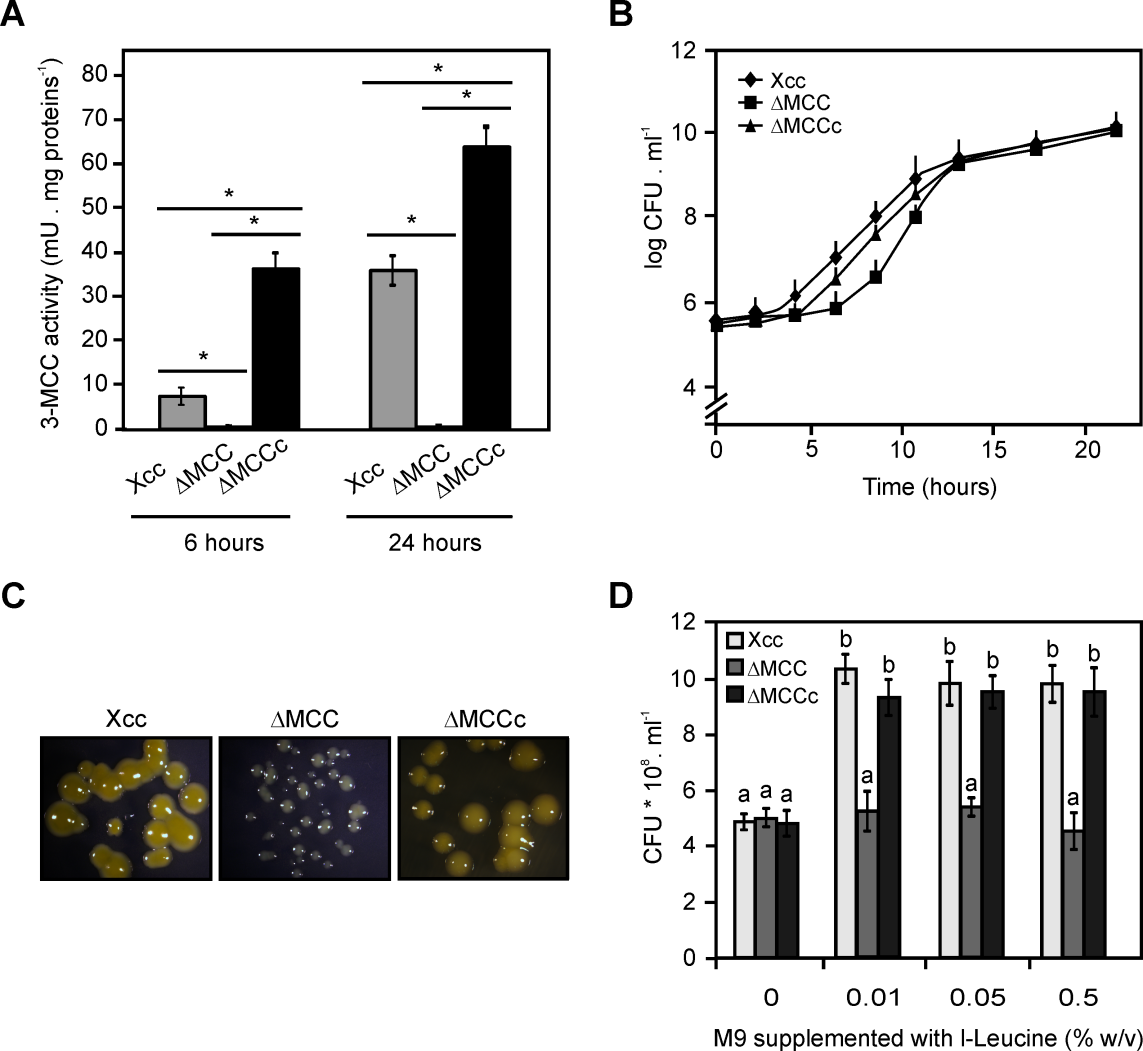
Characterization of MCC mutant. (A) MCC activity measurement in Xcc, ΔMCC and ΔMCCc extracts from bacteria growth at 6 and 24 hours in NB medium. Values are means ± SD of 3 independent experiments. Unpaired t-test was used to determine whether two values were significantly different. *P*-values: *, *P* < 0.05. (B) Bacterial growth of Xcc, ΔMCC and ΔMCCc in NB medium, values represent means of three samples and are representative of three independent experiments. Error bars are standard deviations. (C) Representative images of Xcc, ΔMCC and ΔMCCc grown onto NB plates. (D) Xcc and ΔMCC growth in M9 medium supplemented with different l-Leu concentrations. Bars are the means of 3 samples assayed and error bars are standard deviations, the results are representative of three independent experiments. One-way ANOVA and Tukey post-tests were used to determine whether the values were significantly different. Different letters (a and b) indicate statistically significant differences between groups (mean ± SE). *P*-values: a vs. b, *P* < 0.05.

Expression profiles of *accC and accD* genes *in planta* suggest a role for the Xcc MCC complex in the pathogenicity process. Then, with the purpose of study the physiological function of the MCC complex, we constructed an *accD* mutant in XAC0264 by a single crossover event with the integrative plasmid pK19mobGII, and confirmed the expected mutation by PCR with specific oligonucleotides (see Materials and methods). This strain was named ΔMCC. In order to complement this strain, the region containing *accC and accD* was cloned in-frame in the expression plasmid pBBR1-MCS-5 under the control of the *lacZ* promoter. ΔMCC strain was conjugated with this construction and a resulting transformed complemented-strain was named ΔMCCc. To analyze if the *accD* mutant was impaired in MCC activity, cell-free extracts of Xcc wild-type, ΔMCC and ΔMCCc were prepared at 6 hours at exponential phase of growth, and at 24 hours during stationary phase of growth, in NB medium and assayed for this enzyme activity (Fig 7A). The levels of MCC activity in cell extracts of the ΔMCC mutant were drastically reduced compared to the activity levels found in the wild-type strain. On the other hand, the cell extract of ΔMCCc strain presented a markedly increase in the levels of activity at both time points.

To evaluate the growth rate of the Xcc wild-type, the ΔMCC and the ΔMCCc strains, they were cultured in NB medium for 24 hours and the population of each strain was quantified. Although ΔMCC showed a longer lag phase compared to the wild-type Xcc, all strains reached the same population after 24 hours, demonstrating that in this condition the lack of MCC complex does not impair bacterial growth. The complemented strain showed an intermediate behavior (Fig 7B). However, certain impairment in the growth of the ΔMCC strain was observed as smaller colony size in bacteria cultures grown in solid NB medium after 16 hours (Fig 7C). Then, the growth effect of l-Leu in Xcc and ΔMCC mutant strains was evaluated. Since Xcc could not grow in M9 minimal medium supplemented only with I-Leu as carbon source, this medium was supplemented with 0.4% (w/v) glucose and increasing concentrations of l-Leu (0, 0.01, 0.05 and 0.5% w/v). The presence of l-Leu in this medium promoted an increase in the Xcc growth at all the assayed concentrations (*p* < 0.05) (Fig 7D). On the other hand, the ΔMCC mutant showed no significant differences in bacterial growth in presence of I-Leu (Fig 7D). This result suggests that the MCC complex could provide a metabolic advantage for the Xcc fitness, when this bacterium is growing in a medium containing I-Leu.

### The ΔMCC mutant strain is attenuated in citrus canker disease models

**Fig 8:**
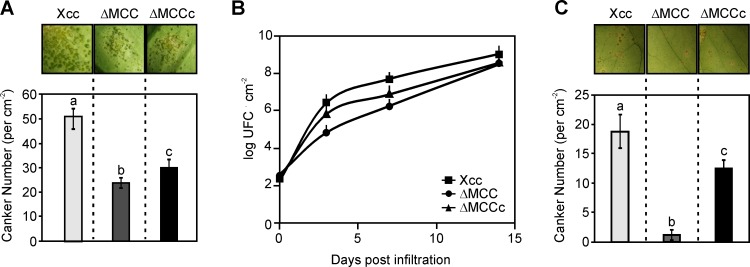
Characterization of plant-pathogen interaction. (A) Fully expanded orange leaves were inoculated at 10^5^ CFU ml^-1^, into the intercellular spaces, with Xcc, ΔMCC or ΔMCCc strains. Representative images are shown 21 days after inoculation (upper panel). In each infiltration, the number of cankers was quantified (bottom). Bars represent the means of 10 leaves assayed, and the standard deviations are showed as error bars. The results are representative of three independent experiments. One-way ANOVA and Tukey post-tests were used to determine whether the values were significantly different. Different letters (a, b and c) indicate statistically significant differences between groups (mean ± SE). *P*-values: a vs. b, *P* < 0.05; a vs. c, *P* < 0.05; b vs. c, *P* < 0.05. (B) Xcc, ΔMCC and ΔMCCc strains were inoculated as in (A), and the bacterial growth in orange leaves were quantified. Values represent means of three samples and are representative of three independent experiments. Error bars correspond to standard deviations. (C) Xcc, ΔMCC and ΔMCCc strains were used to inoculate orange leaves by spraying, at a concentration of 10^9^ CFU ml^-1^. The number of cankers was quantified after one month post spray inoculation. Representative images are shown on the upper panel and on the bottom bars represent the means of 10 leaves assayed. The results are representative of three independent experiments. Standard deviations are indicated as error bars. One-way ANOVA and Tukey post-tests were used to determine whether the values were significantly different. Different letters (a, b and c) indicate statistically significant differences between groups (mean ± SE). *P*-values: a vs. b, *P* < 0.05; a vs. c, *P* < 0.05; b vs. c, *P* < 0.05.

With the aim to evaluate the virulence of the ΔMCC mutant, the host plant *C*. *sinensis* was inoculated with different infection methods. When the infiltration was performed at a concentration of 10^7^ CFU ml^-1^, ΔMCC and wild type strains generate analogous disease signals and no differentiation was observed during the establishment of lesion formation or lesions extensions (S5 Fig). This effect was also observed previously with several Xcc mutants at this high bacterial concentration [52,53]. However, when the leaf tissue was infiltrated with bacteria at a lower concentration (10^5^ CFU ml^-1^) in similar areas, canker numbers (brown spots in Fig 8A, upper) produced by ΔMCC were reduced about 50% (*p* < 0.05) compared to the cankers produced by the wild type strain (Fig 8A, lower). Besides, recovery of bacteria present inside plant tissue during the initial stages of growth, when cankers are still not visible, showed that the population size of the wild type strain was nearly two orders of magnitude higher than the mutant at every time analyzed (3, 7 and 10 dpi) (Fig 8B). In the case of the complemented ΔMCCc strain, an intermediate behavior was observed either in canker numbers or in the bacterial growth inside the plant tissue (Fig 8A and 8B). Furthermore, we also evaluated the virulence of the ΔMCC strain by using a method which simulates the natural infection [54]; for this, the surfaces of the leaves are sprayed with bacteria. One month later, the cankers number was quantified on infected leaves, and resulted 15 times larger (*p* < 0.05) with the wild type strain compared to those obtained with the ΔMCC strain; while the ΔMCCc strain showed a 70% of cankers respect to Xcc (Fig 8C). In order to evaluate if these differences in the virulence of the mutant strains were due to changes in the expression of virulence factors, a qRT-PCR study was performed where the expression of *hrpX*, *hrpB2*, *hrcN*, and *hrcC* genes, involved in the regulation and synthesis of the type III protein secretion system, was analyzed. Bacterial strains were grown 16 h in XVM2 medium and transcript levels analyzed of these genes revealed no differences in genes expression between Xcc, ΔMCC or ΔMCCc strains (S6 Fig). Collectively these findings suggest that the MCC complex from Xcc has a relevant role inside the host cell.

## Discussion

Phytopathogenic bacteria causing major plant diseases are frequently studied in terms of the role of bacterial protein secretion systems, bacterial effector proteins, pathogen associated molecular patterns (PAMPs), and pathogenicity factors in the triggering or overcoming of host defenses. However, infections caused by bacterial phytopathogens involve multiple adaptation processes, such as specific adherence of bacteria to host cells and tissues, and also adaptation of the bacterial metabolism to the nutrients availability and physical conditions existent in host tissues. In this work, we characterized a MCC complex from Xcc at the biochemical and genetic levels, and found that this enzyme, which may be involved in leucine catabolism, is expressed during infection and is necessary for survival into the citrus host tissue.

MCC enzymatic complex belong to the carboxylase superfamily dependent of biotin, having a fundamental role for the catabolism of leucine (S7 Fig). These protein complexes are conformed by two subunits, assembling a α_3_β_6_α_3_ structure of approximately 750 kDa. Their structures were characterized in *Pseudomonas* and humans [34], showing a disposition of two trimers of α subunits, one at each end of a central β_6_ cylindrical core. The biotin-containing component of MCC and GCC were found to have a molecular weight of ~ 75 kDa, which is significantly larger than the ~ 65 kDa α subunit found in those complexes with PCC activity [15,32,34,48]. There exist structural variations in the active site of the MCC and GCC enzymes which justify their differences in the substrate specificity. Precisely, GCC possess a small residue of glycine which is substituted by phenylalanine in MCC, blocking the entry of the larger substrate geranyl-CoA, but admitting the accommodation of 3-methylcrotonyl-CoA. The exchange of these residues by mutation allows the swapping of substrate predilection between the two enzymes [48]. Our bioinformatics analysis pinpoints the presence of two genes, XAC0263 (*accC*) and XAC0264 (*accD*), that encode for the α and β subunits of an ACCase complex, similar to those MCC complexes mainly characterized in, *Pseudomonas*, human and actinomycetes [22,26,34,44]. We also identified that the sequence of Xcc AccD presents a Phe163 residue (equivalent to Phe191 in *P*. *aureginosa*) in the putative catalytic site, suggesting that this complex could holds MCC activity, and that was confirmed biochemically in this study (Fig 4).

ACCases enzymes, including MCC complexes, have essential roles in different metabolic process of most living organisms; however, these enzymes have never been studied in Xcc or any other plant pathogen. The carboxylation of 3-methylcrotonyl-CoA catalyzed by MCC complex is involved in the degradation of the leucine molecule, and other intermediates as isovaleryl-CoA, leading to the generation of acetyl-CoA and acetoacetate [21]. These compounds can be then incorporated or recycled into the metabolism via the TCA cycle and fatty acid biosynthesis.

Sucrose is the main product of photosynthesis in higher plants and is the predominant form of carbohydrate present in the intercellular spaces of citrus leaves along with glucose and fructose [55]. Therefore, these sugars are the main sources of carbon during Xcc infection; however, leucine catabolism can also be significant for the bacterial metabolism as a supplementary or alternative carbon source [56]. Also, in different organisms it was shown that the enzymes involved in leucine catabolism may participate in the detoxification process of noxious intermediates, in regulation and for the reutilization of carbon skeleton of different compounds [56–62].

There are scarce reports related to the characterization of the *in planta* metabolism of Xcc during the pathogenic process, essentially due to important methodological limitations [36,39–42]. Besides, the microenvironment of the bacteria into the plant cells in the different stages of infection remains largely unknown. In order to understand how the pathogen survives and efficiently replicates into the plant tissues, a more extensive aspect of host–pathogen interactions must be evaluated. This includes the complexity of the plant environment, presence of nutrients and the differences of the physiological properties of the pathogen growing in association with a plant compared to those of the pathogen in culture. Moreover, before the internalization into host tissue, during the epiphytic growth, Xcc could be challenged with a complete different environment that can be determinant for the bacterial gene expression and regulation. Considering that the expression levels of *accC* and *accD* were enhanced in the medium that simulates conditions in the plant apoplastic space, and also when bacterial cells were recovered from leaf tissues, it is likely that this complex is involved in the interaction with the host plant. Accordingly, a Xcc ΔMCC mutant was constructed in order to further analyze the role of this complex in virulence. Even if this mutant presented a longer lag phase compared to the wild-type Xcc, both strains reached a similar growth rate during exponential phase and the same population after 24 hours of growth. An interesting difference was observed when the strains were grown in a medium supplemented with leucine; in this case, the growth of the Xcc ΔMCC mutant was not stimulated in the presence of leucine as occurred with Xcc. Therefore, Xcc through the activity of the MCC complex might benefit from the I-Leu catabolism. Overall, these results suggest that the MCC enzymatic complex has an effect on the 3-methylcrotonyl-CoA as part of the l-leucine metabolic pathway in Xcc.

Furthermore, the expression of *accC* and *accD* was induced in the presence of leucine in M9 medium. These results indicate that during the infection process the bacteria could be exposed to a high or moderate concentration of leucine or a related intermediate to its catabolism that stimulates the expression of these genes. In other pathogens, like *M*. *tuberculosis*, the enzyme branched chain keto-acid dehydrogenase, which is also involved in leucine catabolism, was also shown to be required for pathogenesis [61].

Even when Xcc ΔMCC mutant presented a similar growth compared to the wild-type strain in liquid culture, during infection Xcc ΔMCC mutant produced a reduced number of cankers and had a fitness defect compared to the wild type bacteria. These effects were partially compensated when this mutant strain was complemented. So, even if MCC complex activity is probably not directly implicated in the virulence process, it must be necessary for the survival of Xcc during the *in planta* stage. Considering that MCC may be involved in leucine catabolism and intermediates detoxification, it has been observed that in human fibroblasts and in rats, MCC deficiency was recently associated to oxidative stress damage and disruption of energy homeostasis [62,63]. Furthermore, excess of iron levels may lead to the formation of reactive oxygen species in different microorganisms; in *P*. *aeruginosa*, it was shown that the leucine catabolic pathway is regulated by iron through an unknown mechanism [59]. In this context, the concentration of leucine in the plant can change during conditions of stress, development, diurnal/circadian variation, and light availability [64–66], this outcome can impact on the expression levels of MCC complex encoded genes and thus in the performance of Xcc during infection. However, the mechanism of this modulation and the biological relevance still need to be explored. Plant diseases are largely a consequence of molecular interactions between pathogens and their host plants. The metabolic state of *Xanthomonas* during infection has not been studied in details, therefore, the identification and characterization of *in planta* relevant pathways is a priority for investigating new avenues for the control of these phytopathogens.

## Materials and methods

### Bacterial strains, culture and transformation conditions

*Escherichia coli* strain DH5α [67] was used for routine subcloning and it was transformed according to [68]. Transformants were selected on LB media supplemented with the appropriate antibiotics: 50 μg kanamycin (Km) ml^-1^, 20 μg chloramphenicol (Cm) ml^-1^ and 100 μg ampicillin (Ap) ml^-1^.

*E*. *coli* strains harbouring the indicated plasmids (Table 1) were grown at 37°C in Luria Bertani medium (Difco, San Jose, CA).

**Table 1:** Bacterial strains and plasmids.

*Strain*	*Relevant genotype and/or information*	*Source or reference*
Xcc99-1330	*Xanthomonas citri* subsp. *citri*, wild type strain, Ap^r^ (Xcc99-1330)	Inta Bella Vista
ΔMCC	MCC mutant of Xcc99-1330, km^r^ Ap^r^	This study
ΔMCC Complemented	MCC mutant of Xcc99-1330, carries *accC* and *accD* in pBBR1MCS-5 Gm^r^, Km^r^ Ap^r^,	This study
DH5α	*E*. *coli* F^-^ *ϕ*80*lacZ*ΔM15 Δ(*lacZYA*-argF) U169 *endA1 recA1 hsdR17 deoR supE*44 *thi-1 gyrA96 relA1*	Laboratory stock
BL21 λ(DE3)	*E*. *coli* F^-^ *ompT rB*^*-*^ *mB*^*-*^ λ(DE3), pLysS, Cm^r^.	[69]
S17-1	TpR SmR *recA thi pro hsdR*-M+RP4: 2-Tc:Mu: Km Tn7 λ*pir*.	[70]
*Plasmids*		
pCR-Blunt II-TOPO^®^	Cloning Vector, Km^r^	Invitrogen
pLS18	pET28a(+) derivate (Km^r^ *lacZ*’) for expression of recombinant proteins under control of strong T7 transcription and translation signals	Lautaro Diacovich & Salvador Peirú
pCY216	Vector containing *E*. *coli birA* gene	[71]
pBBR1-MCS5	Broad host-range vector, Gm^r^	[72]
pMT1	pCR-Blunt II-TOPO^®^ derivative carrying *accD gene*	This study
pMT2	pLS18 derivative carrying *accD* gene	This study
pMT3	pCR-Blunt II-TOPO^®^ derivative carrying *accC* gene	This study
pMT4	pLS18 derivative carrying *accC* gene	This study
pMT5	pLS18 derivative carrying both *accC* and *accD* cloned in frame	This study
pMTcompDC	pBBR1-mcs5 derivative carrying *accD* and *accC* genes cloned in frame	This study
pK19mobGII	pUC19 derivative, *lacZa*, *gusA*, mob *site*, Km^r^	[73]
pK19INTaccD	pK19mobGII carrying an internal *accD* region	This study

*Xanthomonas citri* subsp. *citri* (Xcc) wild-type strain Xcc99-1330 [52] and mutant strains were grown at 28°C in Nutrient Broth (NB) medium (3 g/l meat extract, 5 g/l peptone, pH 7.0), XVM2 medium (20 mM NaCl, 10 mM (NH_4_)_2_SO_4_, 1mM CaCl_2_, 0.01 mM FeSO_4_, 5 mM MgSO_4_, 0.16 mM KH_2_PO_4_, 0.32 mM K_2_HPO_4_, 10 mM fructose, 10 mM sucrose and 0.03% casein acid hydrolysate (casaminoacid), pH 6.7) [47], or M9 (25 mM KH_2_PO_4_, 50 mM NaH_2_PO_4_.7H_2_O, 10 mM NaCl, (NH_4_)_2_SO_4_ 1.2 g/l, 1 mM MgSO_4_, 0.2 mM CaCl_2_ and 0.4% (w/v) glucose. l-Leucine was added at different final concentrations between 0.01% and 0.5% w/v, in M9 liquid medium. The appropriate antibiotics were used at the following final concentrations: 25 μg ampicillin (Ap) ml^-1^, 20 μg gentamicin (Gm) ml^-1^ and 25 μg kanamicin (Km) ml^-1^. To evaluate the bacterial growth in presence of l-leucine in M9 medium, Xcc was grown in NB until exponential growth phase, cells were recovered by centrifugation, washed and inoculated in fresh M9 medium supplemented or not with 0.01, 0.05 and 0.5% (w/v) l-leucine during 16 h. In all cases 0.4% w/v glucose was used as carbon source. Bacterial serial dilutions were plated onto NB agar plates, and colonies counted after 48 h of incubation at 28°C. The survival average was calculated relative to the control in M9 without l-leucine.

### DNA manipulations

Isolation of plasmid DNA, restriction enzyme digestion and agarose gel electrophoresis were carried out by conventional methods [68].

### Gene cloning and plasmid construction

In all cases, the template for the PCRs was genomic DNA from wild-type *Xcc*. The cloning vector used was pCR-Blunt II-TOPO^®^. It allows direct insertion of PCR products, which were amplified by using the synthetic primers listed in Table 2. A 1680 bp fragment containing *accD* ORF was cloned into pCR-Blunt II-TOPO^®^ to generate pMT1. This plasmid was digested with restriction enzymes *Nde*I and *Spe*I and the restriction fragment was inserted into previously digested pLS18 to generate pMT2.

**Table 2:** Oligonucleotides used in this study.

*Oligonucleotide*	*Sequence (5’ to 3’)*	*Restriction site*
accCup	TGAT**CATATG**ACCCAGCGCGAC	*Nde*I
accCdn	TGGT**AAGCTT**GC**ACTAGT**CTGCTACTACGCAGACG	*Hind*III, *Spe*I
accDup	AGCC**CATATG**AGCGTGATCGATAG	*Nde*I
accDdn	TG**AAGCTT**GC**ACTAGT**ACCTCACATGCGAAACAC	*Hind*III, *Spe*I
accDCup	CC**CATATG**AGCGTGATCGATAGCCAGC	*Nde*I
accDCdn	CT**AAGCTT**AG**ACTAGT**CGCAGGCGAACGTGC	*Hind*III, *Spe*I
accDF	CGC**GGATCC**TGACGGTGAAGAAGCATTTG	*Bam*HI
accDR	CCC**AAGCTT**AAAACAGGATGCCGTTGTTG	*Hind*III
accCRT-F	GTTACCACGGTGACGAAC	
accCRT-R	ACATATTTTTCCACCAGCAC	
accDRT-F	TCTTCTACAACCAGGCCAAT	
accDRT-R	CAGGTCTTCAGCACTGACTT	
HrpXRT-F	CGATGATGAGGTCAGTTTGT	
HrpXRT-R	ACTGCGCAAAGCAATTCAAC	
HrcCRT-F	TTCGTCTGGTACTACGATGG	
HrcCRT-R	CCGAAACGGTATCCACATAC	
hrpB2RT-F	AACCAAGCGCTTGTGAATCG	
hrpB2RT-R	CTATTGGTTCTTGACCAGTG	
hrcNRT-F	GAACCAGTACCCGGCAATC	
hrcNRT-R	GTCGGTTGGCTGAGAAAGTC	
16S rRNA up	TGGTAGTCCACGCCCTAAACG	
16S rRNA down	CTGGAAAGTTCCGTGGATGTC	
ActinL	ACGTGAATTCTAGTGTTTCGATAAGT	
ActinR	TCAATTGGATACTTCAAAGTCAAAAT	

The same procedure was performed to clone a 2100 bp DNA fragment containing *accC* ORF into pCR-Blunt II-TOPO^®^, generating pMT3. pMT3 was digested with *Nde*I and *Spe*I and the resulting restriction fragment was cloned into pLS18 to give rise to pMT4. pMT5 vector was generated by inserting *accD* fragment *(Hind*III, *Spe*I) from pMT2 into pMT4 previously digested with restriction enzymes *Hind*III and *Xba*I. This procedure creates a construction in which *accC* and *accD* genes are cloned in tandem and in frame, transcriptionally fused to poly His tags, and under T7 promoter control. The same construct was extracted from pMT5 with *Spe*I and *Bgl*II restriction enzymes and cloned into pBBR1mcs5 vector previously digested with *Spe*I and *Bam*HI, thus creating pMTcompDC.

### Protein expression and purification and protein methods

For the expression of heterologous recombinant *Xanthomonas* His-tagged proteins, *E*. *coli* strains harboring the appropriate plasmids (pMT2 (His_6_-AccD), pMT4 (His_6_-AccC), or pMT5 (His_6_-AccD and His_6_-AccD) were grown at 37°C in shake flasks in Luria-Bertani (LB) medium in the presence of the corresponding antibiotics for plasmid maintenance. Overnight cultures were diluted 1:100 in fresh medium and grown to an *A*600 of 0.5 to 0.8 before the addition of IPTG to a final concentration of 0.5 mM [69] and also 0.5% arabinose was added for the case of biotin ligase induction. Induction was allowed to proceed overnight at 20°C. The cells were harvested, washed, and resuspended in buffer A (50 mM Tris-HCl, pH 8, 300 mM NaCl, 0.75 mM dithiothreitol [DTT], 1 mM EDTA, 10% [v/v] glycerol). Cells were resuspended in buffer A and disrupted by sonication, and the lysate was clarified by centrifugation at 20,000 g and 4°C for 30 min. The supernatant was applied to a Ni^2+^ (NTA)-agarose affinity column (QIAGEN), equilibrated with the same buffer supplemented with 20 mM of imidazole. The column was subsequently washed, and the His_6_-tagged proteins were eluted from the column using binding buffer containing 0 to 250 mM imidazole. Fractions of the eluate were collected and analyzed for protein by SDS-PAGE [74] using a Bio-Rad mini-gel apparatus. The final acrylamide monomer concentration was 10% (w/v) for the separating gel and 5% for the stacking gel. Coomassie brilliant blue was used to stain protein bands. The fractions containing purified proteins were dialyzed at 4°C overnight against 100 mM potassium phosphate, pH 7.6, 0.75 mM DTT, 1 mM EDTA, and 20% glycerol (v/v). Proteins were stored at -80°C. To improve the biotinylation of AccC in *E*. *coli*, the strains containing pMT4-5 were also transformed with pCY216 [71], which overexpresses the *E*. *coli* biotin ligase (BirA); 10 μM D-biotin was also added to the medium.

Protein contents were determined by measuring its A280nm, by the method of Bradford [75] with bovine serum albumin as a standard, and QubitR fluorometer (Invitrogen).

The biotinylated protein (AccC) was detected by a modification of the Western blotting procedure described by Nikolau et al. [76]. After electrophoretic separation, proteins were electroblotted onto nitrocellulose membranes (Bio-Rad) and probed with alkaline phosphatase (AP)-streptavidin conjugate (diluted 1:5,000) (Bio-Rad).

### Size exclusion chromatography

Molecular mass of each subunit and the assembled MCC complex was estimated by size exclusion chromatography using an AKTÄ basic high-performance liquid chromatograph (GE). Samples containing 500 μg of AccC, AccD or the complex were loaded onto a Superdex S200 column (GE). The column was equilibrated in 50 mM potassium phosphate, pH 7.6, 50 mM NaCl, and 0.5 mM DTT and eluted with the same buffer. Absorbance at 280 nm was recorded. The column was calibrated with the following molecular mass standards: Carbonic Anhydrase; Bovine Erythrocytes (29000); Albumin, Bovine Serum (66000); Alcohol Dehydrogenase, Yeast (150000); β-Amylase, Sweet Potato (200000); Apoferritin, Horse Spleen (443000); Thyroglobulin, Bovine (669000); and blue dextran (2000000).

### ACCase enzyme assays

ACCase activities in cell-free extracts and with the purified complex were measured by following the incorporation of radioactive HCO_3_^-^ into acid non-volatile material, as previously described [50]. Substrate concentrations were 0.5 mM for acetyl-, propionyl-, butiryl- and 3-methylcrotonyl-CoA. One unit of enzyme activity catalyzed the incorporation of 1 mmol ^14^C into acid-stable products min^-1^. A reaction in presence of 3-methylcrotonyl-CoA as a substrate and without cell extract was used as a control; the very low basal activity value obtained for this reaction was subtracted to those values obtained for all the experiments used in this study.

To evaluate MCC activity in Xcc, ΔMCC and ΔMCCc extracts, bacterial strains were grown at 28°C in Nutrient Broth (NB) at 4, 6, 8 and 24 h. Bacteria were harvested by centrifugation and cells were resuspended in buffer 100 mM potassium phosphate, pH 7.6, 0.75 mM DTT, 1 mM EDTA, and 20% glycerol (v/v) and disrupted by sonication, and the lysate was clarified by centrifugation at 20,000 g and 4°C for 30 min. 25 μg from the supernatant fraction was used to performed ACCase assay.

Pyruvate kinase-lactate dehydrogenase (PK-LDH) assay: The rate of ATP hydrolysis by biotin carboxylase was measured spectrophotometrically [10]. The production of ADP was coupled to PK and LDH, and the oxidation of NADH was monitored at 340 nm [77]. Assays were performed in a Synergy2 microplate reader as previously described [50]. Under the assay conditions described, the reaction was linear for at least three min and the initial rate of reaction was proportional to the enzyme concentration. Initial velocities were obtained from initial slopes of the recorder traces. One unit of enzyme activity catalyzes the formation of 1 mmol of the respective carboxylated CoA derivative or ADP min^-1^ under the assay conditions described. Specific activity is expressed as units per mg of MCC complex, considering it as a heterododecamer. The kinetic parameters of the MCC complex for the short chain acyl-CoAs were obtained with the method described above but varying the 3-methylcrotonyl-CoA (Sigma) concentrations between 1 and 1000 μM. The reaction was carried out in the presence of 0.05 μM of the enzyme complex.

### RNA preparation and RT-PCR

Xcc cells were grown until stationary phase for the analysis of *accC* (XAC0263) and *accD* (XAC0264) expression in NB or XVM2 media. To analyze the gene expression in presence of l-leucine in M9 medium, Xcc was grown in NB until exponential growth phase, later cells were recovered by centrifugation, washed and inoculated in fresh M9 medium supplemented or not with 0.5 and 1% (w/v) l-Leu during 1 hour. In all cases 0.4% w/v glucose was used as carbon source. RNA preparations from bacteria recovered from inoculated leaves at 0 and 3 days post infection were done as described previously [52,78]. Briefly, 20 citrus leaves were inoculated with Xcc and 10 leaves at each time of infection were harvested and immediately sliced into thin pieces with a sterile razor blade and maintained for 1 h in sterile glass plates containing 15 ml of distilled water for bacterial exudation. The leaves were separated from the suspension by pipetting the water, which was centrifuged to pellet the bacterial cells. In the case of transcript analysis of *hrpX*, *hrpB2*, *hrcN and hrcC* genes, Xcc, ΔMCC or ΔMCCc strains were cultures in XVM2. Total RNA was extracted immediately using TriPure Isolation Reagent (Roche) according to the manufacturer’s instructions. After treatment with DNAse (Promega), cDNA was synthesized from 2 μg of total RNA using MMLV RT (Promega) and the oligonucleotide dN6. To detect any plant RNA contamination the pair of oligonucleotides: ActinL and ActinR (Table 2) that amplified a fragment of 800-bp of the plant actin gene were used in a similar PCR reaction. To analyze the expression of *accC* and *accD*, PCR was done with 0.05 μg cDNA template using the following pairs of oligonucleotides: accCRT-F and accCRT-R, and accDRT-F and accDRT-F (Table 2) under the following conditions: 94°C for 2 min, followed by 27 cycles of 94°C for 30 sec, 55°C for 30 sec, and 72°C for 30 sec, and final extension at 72°C for 10 min. As a constitutive control a 217 bp fragment of 16S rRNA was amplified using the same PCR conditions with the pair of oligonucleotides: 16S rRNA up and 16S rRNA down (Table 2), which does not modify its expression in different growth condition analyzed [79,73]. PCR products were electrophoresed in a 2% (w/v) agarose gel and photographed with FOTO/Analyst® Investigator Eclipse® (BioRad) and Gel-Pro Analyzer Software 3.1 (Media Cybernetics) were used to measure the intensity of each band. qRT-PCR of *hrp* genes were performed in a Mastercycler ep realplex thermal cycler (Eppendorf) using SYBR Green I (Roche) as described [52].

### Generation of MCC insertional mutant

A 693-bp internal region of *accD* (XAC0264) was amplified by PCR using a pair of oligonucleotides. The pair used for the amplification of this region was accDF and accDR (Table 2), containing the restriction sites for *Bam*HI and *Hind*III, respectively. Genomic DNA (100 ng) was used as the template in PCR (50 μl reaction volume) performed in an Ivema T-18 thermal cycler, with denaturation at 94°C for 3 min, followed by 30 cycles of 94°C for 1 min, 58°C for 1 min, and 72°C for 2 min, and final extension at 72°C for 10 min. Amplified product of the internal *accD* region previously digested with *Bam*HI and *Hind*III was cloned in pK19mobGII [73] digested with the same restriction enzymes, rendering pK19INTaccD. *E*. *coli* S17-1 cells [70] transformed with pK19INTaccD were conjugated to Xcc and selected for km resistance to obtain ΔMCC insertional mutant by a simple recombination event. The Xcc ΔMCCc complemented strain was constructed by cloning *accC* and *accD* from pMT5 in the replicative plasmid pBBR1MCS-5 [72].

### Plant material and inoculations

Orange (*Citrus sinensis* cv. valencia) was used as the host plant for Xcc. Plants were grown in a growth chamber in incandescent light at 28°C with a photoperiod of 16 h. Bacteria were cultured in NB broth to an optical density at 600 nm (OD_600_) of 1, harvested by centrifugation, and resuspended in 15 mM NaCl at 10^5^ to 10^7^ CFU ml^-1^. For disease symptoms assays, bacterial suspensions were infiltrated into leaves with needleless syringes [80]. *In planta* growth assays were performed by grinding 0.8 cm diameter leaf discs from infiltrated leaves in 1 ml of 15 mM NaCl, followed by serial dilutions, and plating onto NB agar plates. Colonies were counted after 48 h of incubation at 28°C, and the results are presented as CFU cm^-2^ of leaf tissue. Cankers were count from 10 orange leaves infiltrated with 10^5^ CFU ml^-1^ and the areas of the counted leaves were measured from digitalized images using Adobe Photoshop software. Epiphytic fitness was evaluated through bacterial inoculations at 10^9^ CFU ml^-1^ in 15 mM NaCl, by spraying on orange leaves until both leaf surfaces were uniformly wet. Canker numbers per cm^2^ of leaf tissue were counted after 1 month post inoculation.

## Supporting information

S1 Table Porcentaje of identity of α (A) and β (B) subunits from different organisms.
(TIF)

S1 Fig Sequence alignment of the putative α subunits of different α-proteobacteria. Residues with important functions are highlighted in color, using as a reference the MCCα from *P*. *aeruginosa*. Blue, residues involved in ATP binding; black, residues making up the active site; red, residues of lysine and cysteine having a role in the catalysis; green, aminoacidic background involved in biotin binding. XCC, *Xanthomonas*; Psa, *Pseudomonas aeruginosa*; Geo, *Geobacter picjeringii*; Bor, *Bordetella pertusis*; Ba, *Brucella abortus*.
(TIF)

S2 Fig Sequence alignment of the putative β subunits of different α-proteobacteria. Residues with important functions are highlighted in color, using as a reference the MCCβ from *P*. *aeruginosa*. Blue, residues involved in coenzyme A binding; red, residues forming the pocket to stabilize one of the γ carbons of the molecule substrate; green, BCCP binding domain; black, highly conserved residues forming the oxyanion. XCC, *Xanthomonas*; Psa, *Pseudomonas aeruginosa*; Geo, *Geobacter picjeringii*; Bor, *Bordetella pertusis*; Ba, *Brucella abortus*.
(TIF)

S3 Fig Purification and analysis of MCC subunits from Xcc. (A) Purification of AccD. (B) Purification of AccC. (C) Purification of AccC-AccD complex. Each His-tagged protein was purified as described in Materials and Methods section. Elution fractions were collected, dialyzed and used for further experiments; 10% Tris/glycine SDS/PAGE was used. PF, pellet fraction; FT, flow through; MWM, molecular weight marker; WS, wash; E, elution. (D) Western blot analysis of purified fractions. Fractions E5 y E6 from panel C were run on SDS-PAGE, transferred to nitrocellulose, and probed with alkaline phosphatase-streptavidin conjugate.
(TIF)

S4 Fig Bacterial growth of XCC in NB medium. Wild type Xcc strain was growth at 28°-C in NB medium and followed by measuring OD_600 nm_. Values represent means of three samples and are representative of three independent experiments. Error bars are standard deviations. Arrows indicate the times when aliquots of the cultures were collected for further analysis (T1, T2, T3 and T4).
(TIF)

S5 Fig Characterization of plant-pathogen interaction. Citrus leaves were inoculated with Xcc, ΔMCC or ΔMCCc strains at 10^7^ CFU ml^-1^ in 15 mM NaCl. A representative image of lesions is shown 7 days after inoculation.
(TIF)

S6 Fig Expression of virulence genes in Xcc, MCC mutant and complemented strains. qRT-PCR analysis of *hrpX*, *hrcC*, *hrpB2* and *hrcN* gene expression using total RNA obtained from Xcc, mutant ΔMCC and ΔMCCc bacterial strains grown in XVM2 medium. As a reference the amplification of a fragment of 16S rRNA gene was used. Values represent the means of three independent experiments. Error bars indicate standard deviations. Data were statistically analyzed using one-way ANOVA. *P-value* < 0.05.
(TIF)

S7 Fig Pathway of l-leucine catabolism in bacteria. The reactions catalyzed by the enzymes are represented by arrows. The metabolism of the amino acid leucine requires 3-MCC activity (highlighted). The enzymes are: leucine transaminase, 2-ketoisocaproic dehydrogenase, isovaleryl-CoA dehydrogenase, 3-methylcrotonyl-CoA carboxylase, 3-methylglutaconyl-CoA hydratase, 3-hydroxy-3-methylglutaryl-CoA lyase.
(TIF)
