# Supplementary figures and images for: 3-methylcrotonyl Coenzyme A (CoA) carboxylase complex is involved in the Xanthomonas citri subsp. citri lifestyle during citrus infection
Source: PLoS One. 2018 Jun 7;13(6):e0198414. doi: 10.1371/journal.pone.0198414 (PMC5991677; doi:10.1371/journal.pone.0198414)

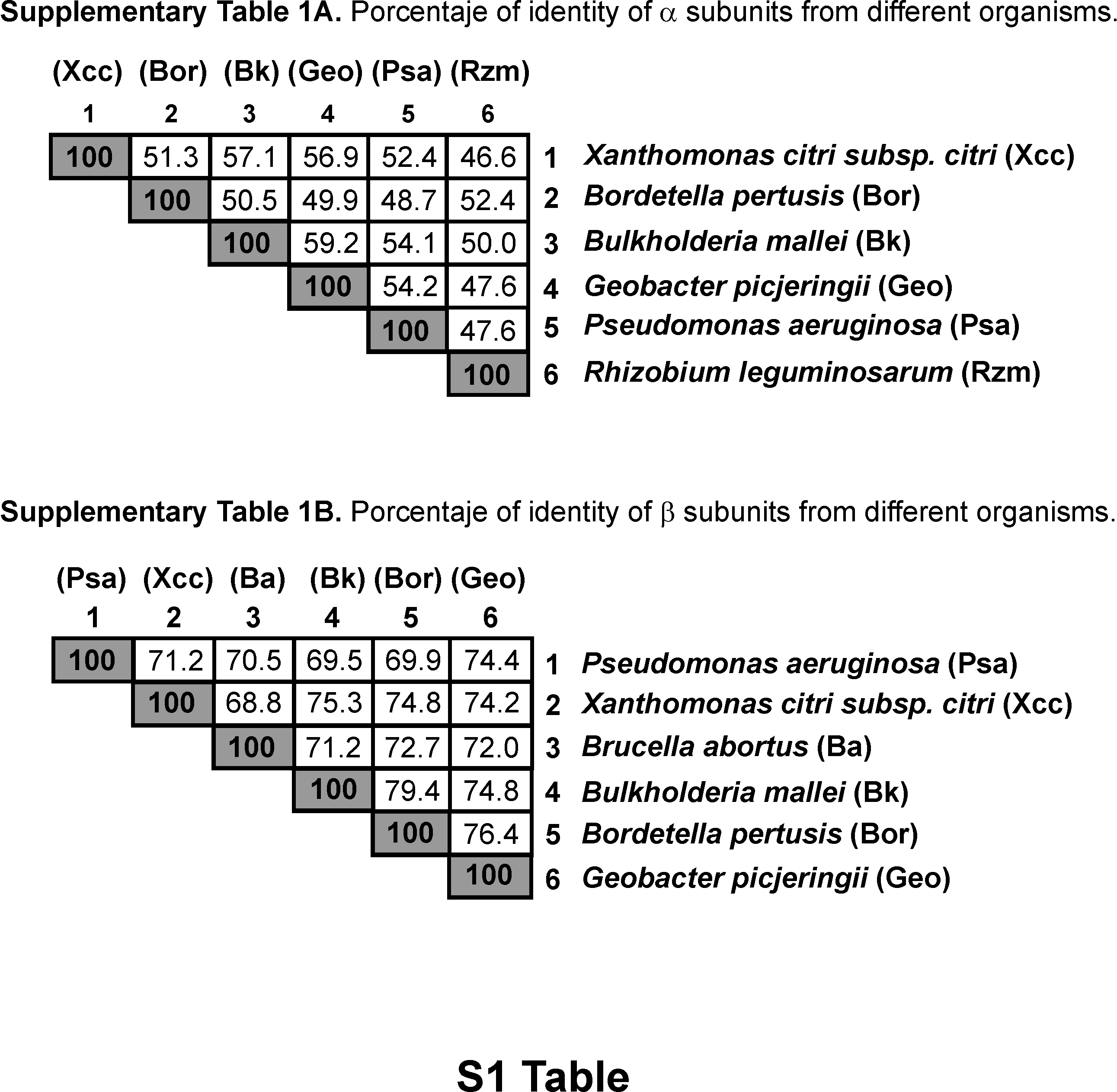

Supplement: S1 Table — Porcentaje of identity of α (A) and β (B) subunits from different organisms. (TIF) [file pone.0198414.s001.tif]

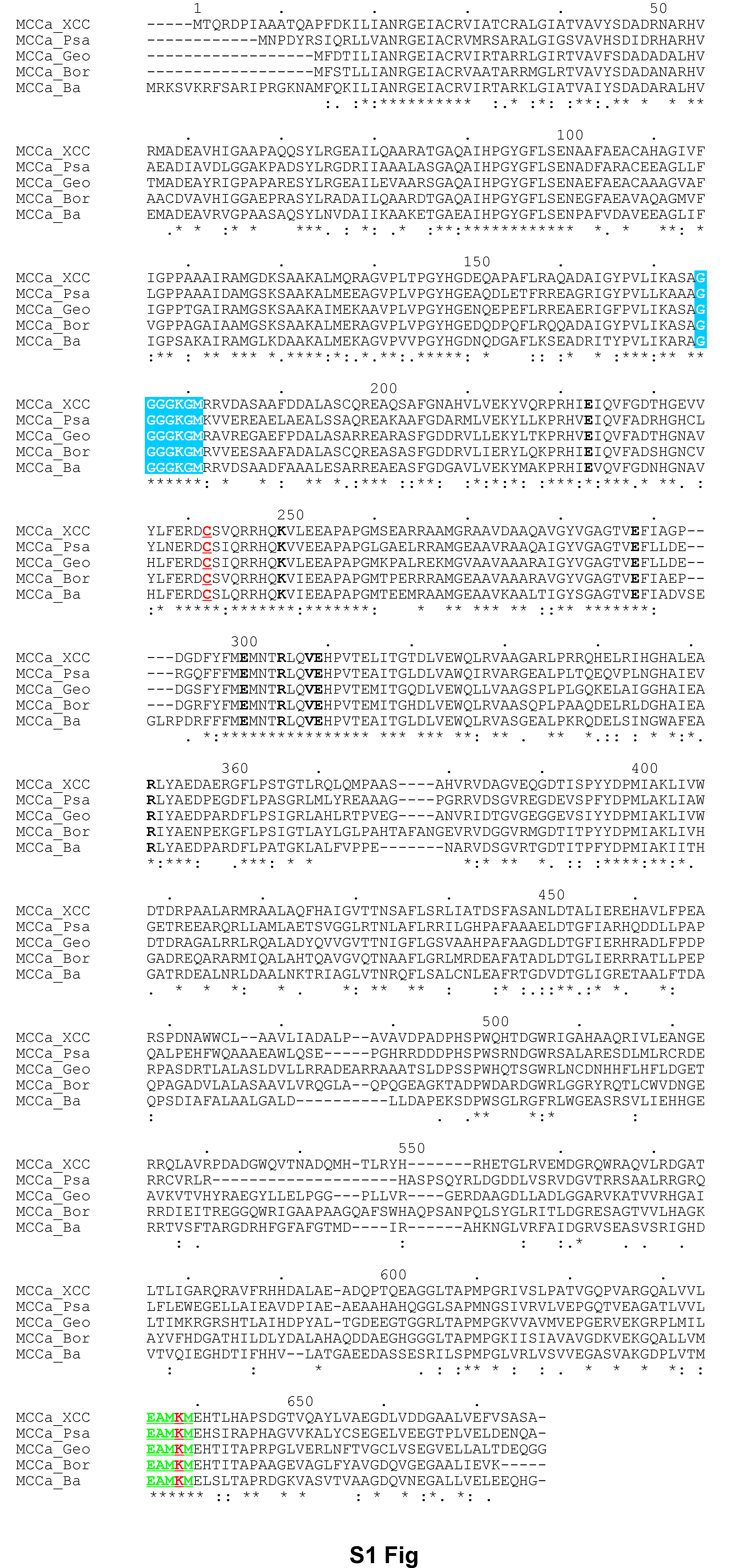

Supplement: S1 Fig — Residues with important functions are highlighted in color, using as a reference the MCCα from P. aeruginosa. Blue, residues involved in ATP binding; black, residues making up the active site; red, residues of lysine and cysteine having a role in the catalysis; green, aminoacidic background involved in biotin binding. XCC, Xanthomonas; Psa, Pseudomonas aeruginosa; Geo, Geobacter picjeringii; Bor, Bordetella pertusis; Ba, Brucella abortus. (TIF) [file pone.0198414.s002.tif]

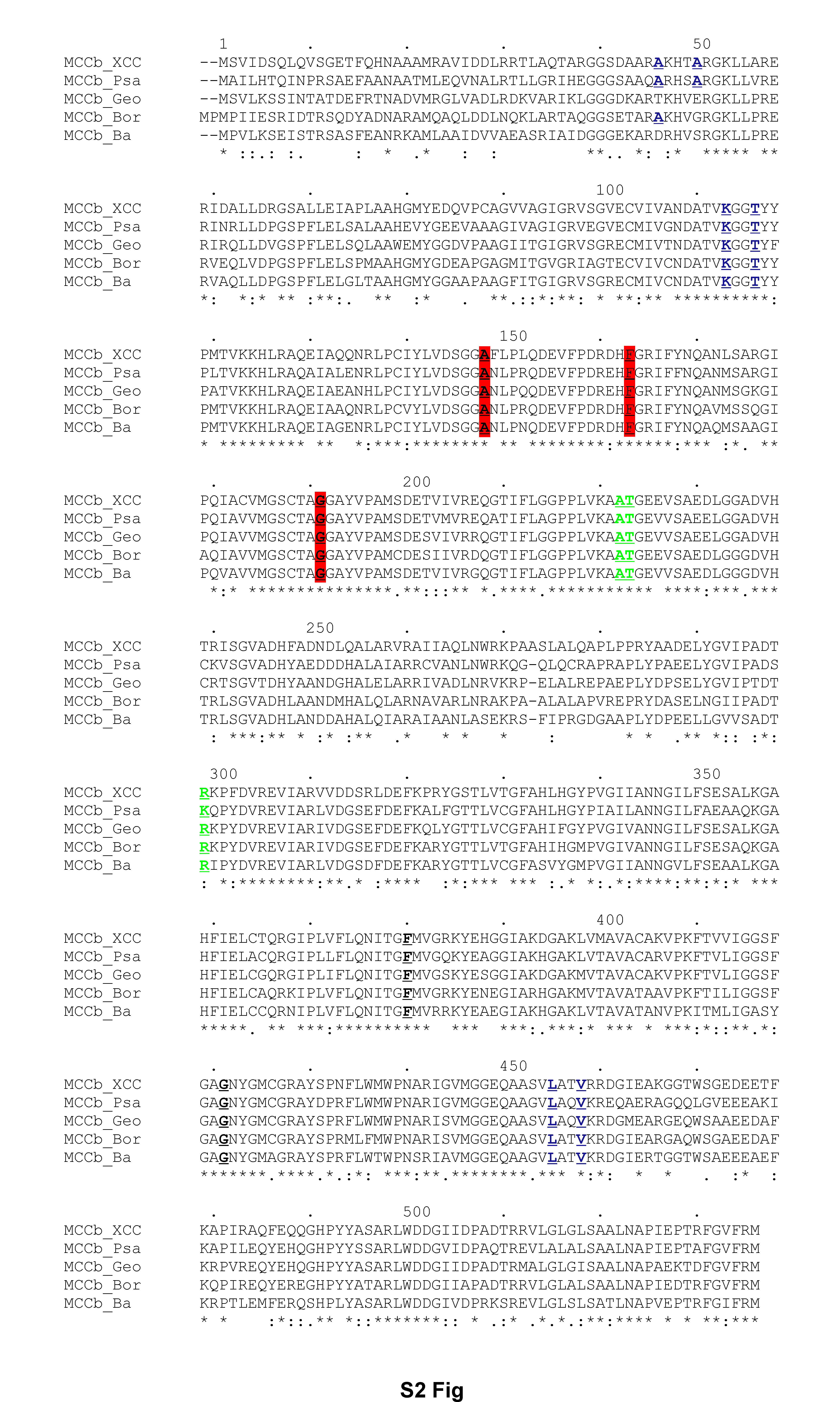

Supplement: S2 Fig — Residues with important functions are highlighted in color, using as a reference the MCCβ from P. aeruginosa. Blue, residues involved in coenzyme A binding; red, residues forming the pocket to stabilize one of the γ carbons of the molecule substrate; green, BCCP binding domain; black, highly conserved residues forming the oxyanion. XCC, Xanthomonas; Psa, Pseudomonas aeruginosa; Geo, Geobacter picjeringii; Bor, Bordetella pertusis; Ba, Brucella abortus. (TIF) [file pone.0198414.s003.tif]

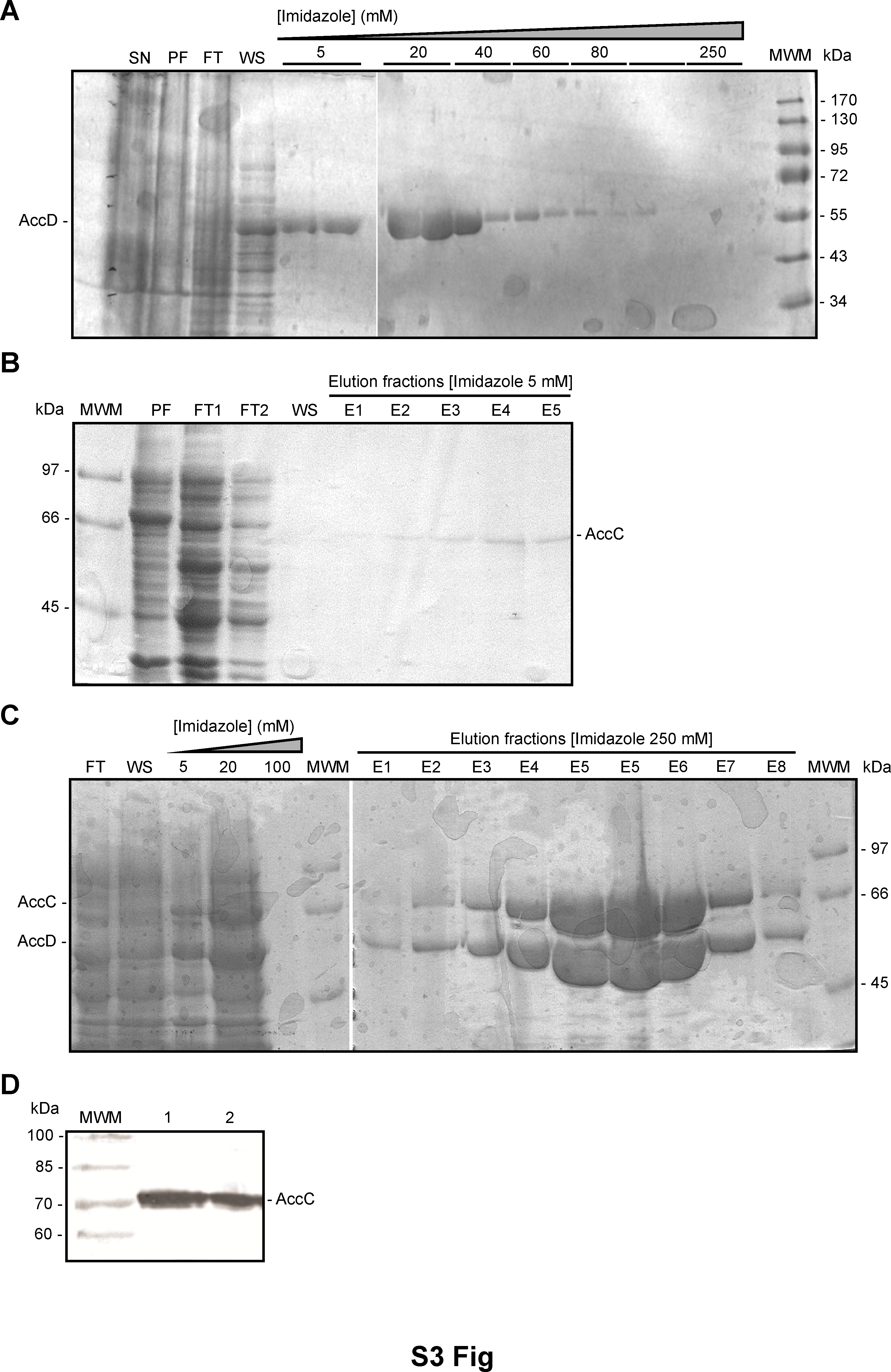

Supplement: S3 Fig — (A) Purification of AccD. (B) Purification of AccC. (C) Purification of AccC-AccD complex. Each His-tagged protein was purified as described in Materials and Methods section. Elution fractions were collected, dialyzed and used for further experiments; 10% Tris/glycine SDS/PAGE was used. PF, pellet fraction; FT, flow through; MWM, molecular weight marker; WS, wash; E, elution. (D) Western blot analysis of purified fractions. Fractions E5 y E6 from panel C were run on SDS-PAGE, transferred to nitrocellulose, and probed with alkaline phosphatase-streptavidin conjugate. (TIF) [file pone.0198414.s004.tif]

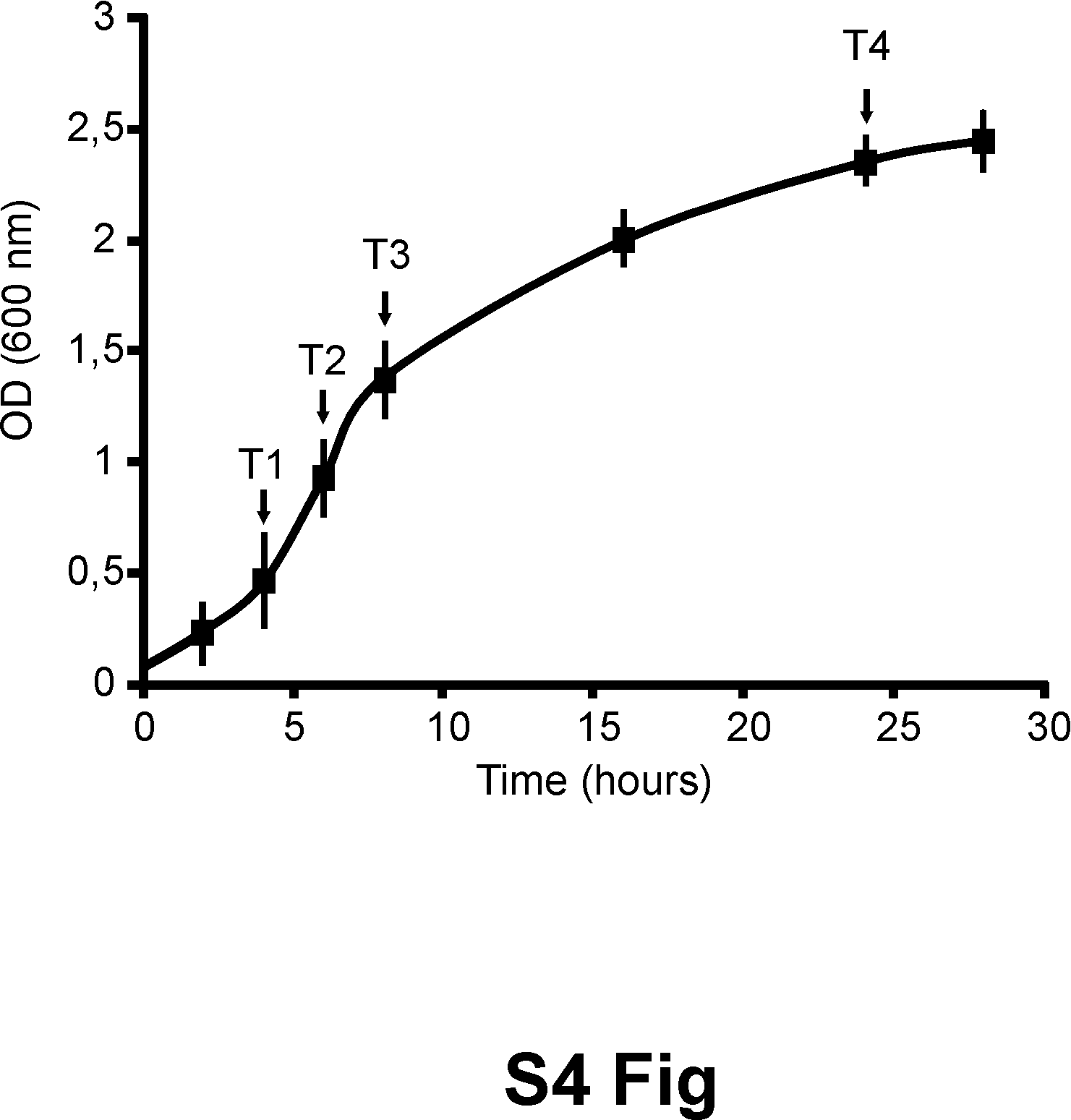

Supplement: S4 Fig — Wild type Xcc strain was growth at 28°-C in NB medium and followed by measuring OD600 nm. Values represent means of three samples and are representative of three independent experiments. Error bars are standard deviations. Arrows indicate the times when aliquots of the cultures were collected for further analysis (T1, T2, T3 and T4). (TIF) [file pone.0198414.s005.tif]

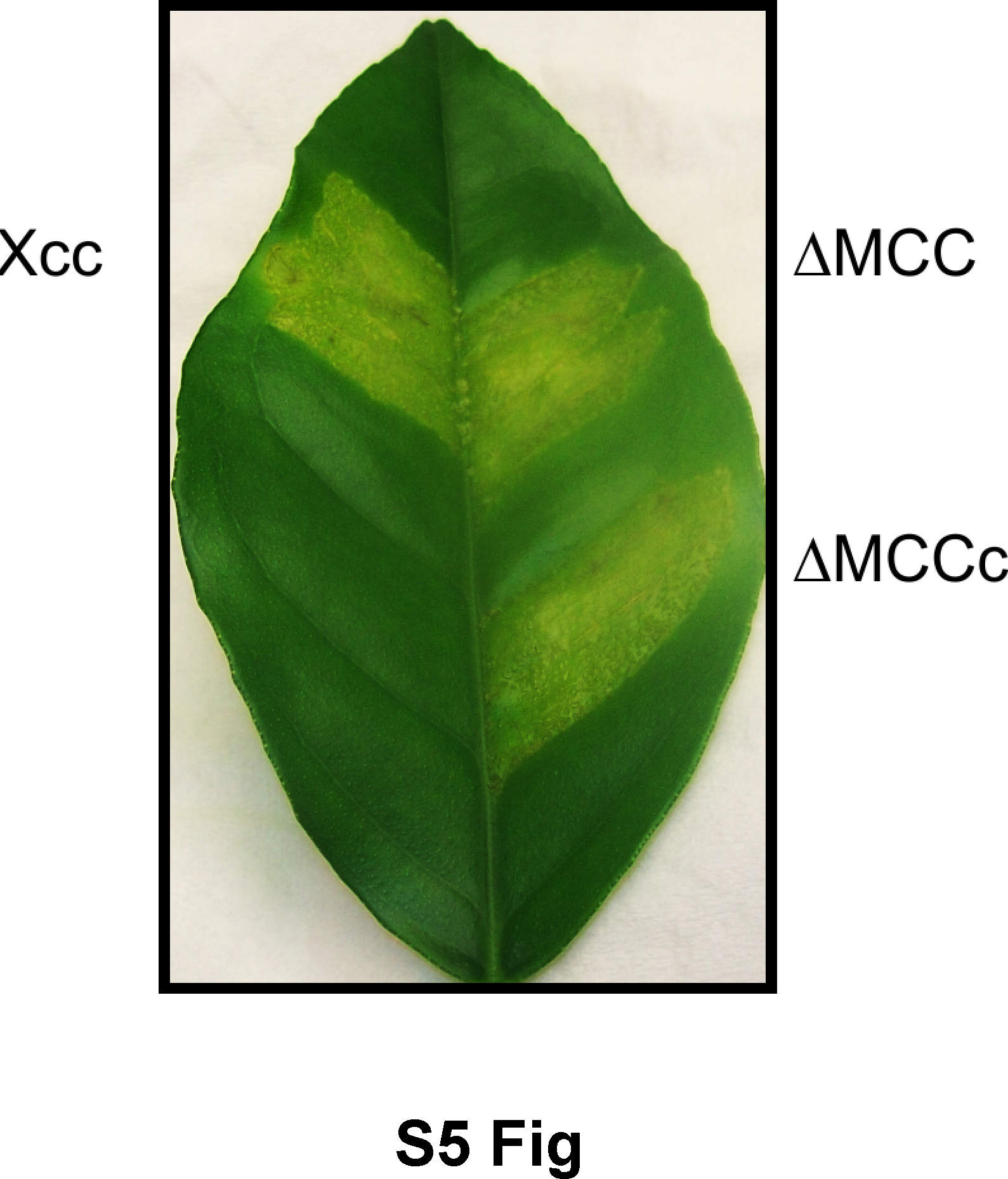

Supplement: S5 Fig — Citrus leaves were inoculated with Xcc, ΔMCC or ΔMCCc strains at 107 CFU ml-1 in 15 mM NaCl. A representative image of lesions is shown 7 days after inoculation. (TIF) [file pone.0198414.s006.tif]

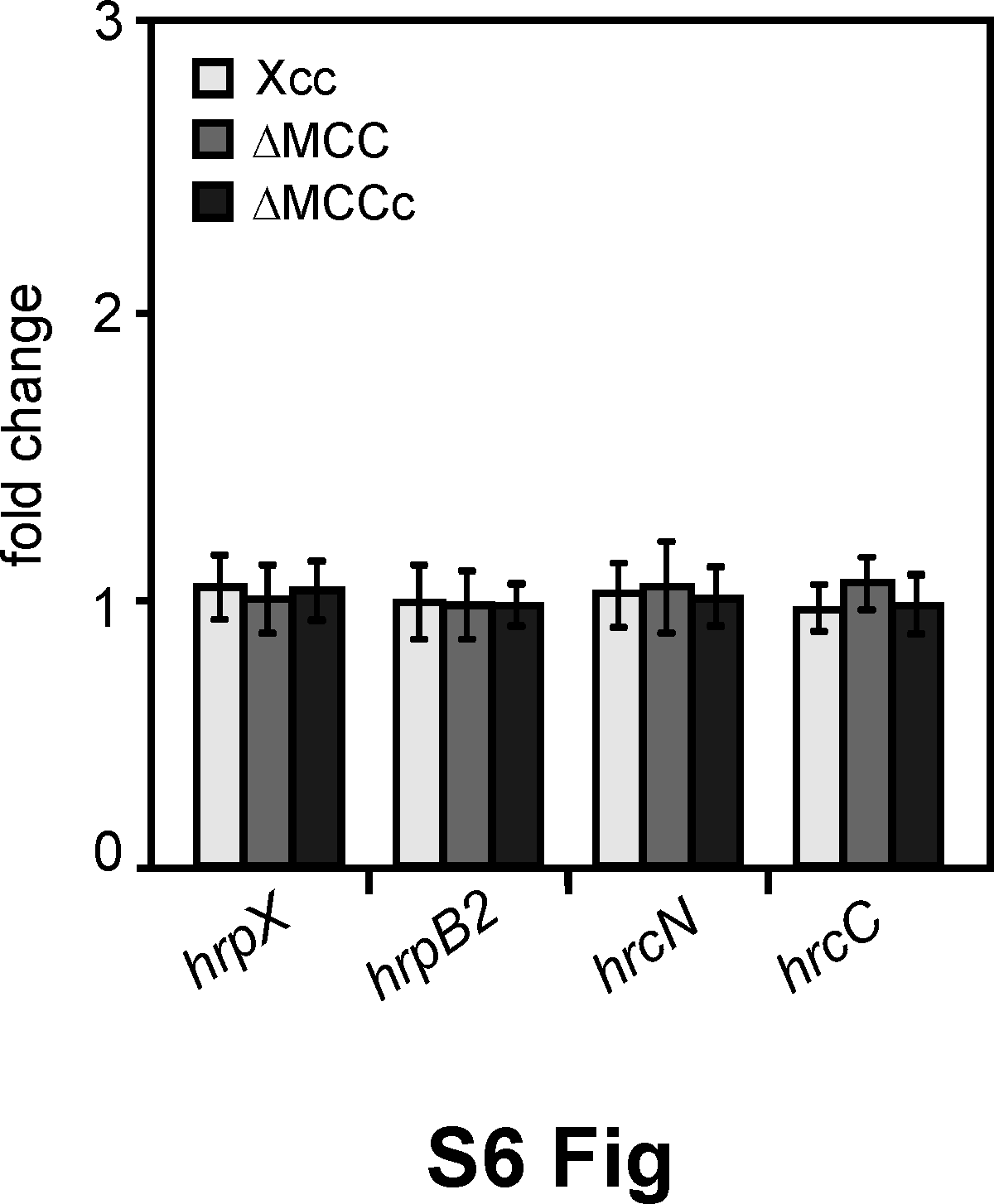

Supplement: S6 Fig — qRT-PCR analysis of hrpX, hrcC, hrpB2 and hrcN gene expression using total RNA obtained from Xcc, mutant ΔMCC and ΔMCCc bacterial strains grown in XVM2 medium. As a reference the amplification of a fragment of 16S rRNA gene was used. Values represent the means of three independent experiments. Error bars indicate standard deviations. Data were statistically analyzed using one-way ANOVA. P-value < 0.05. (TIF) [file pone.0198414.s007.tif]

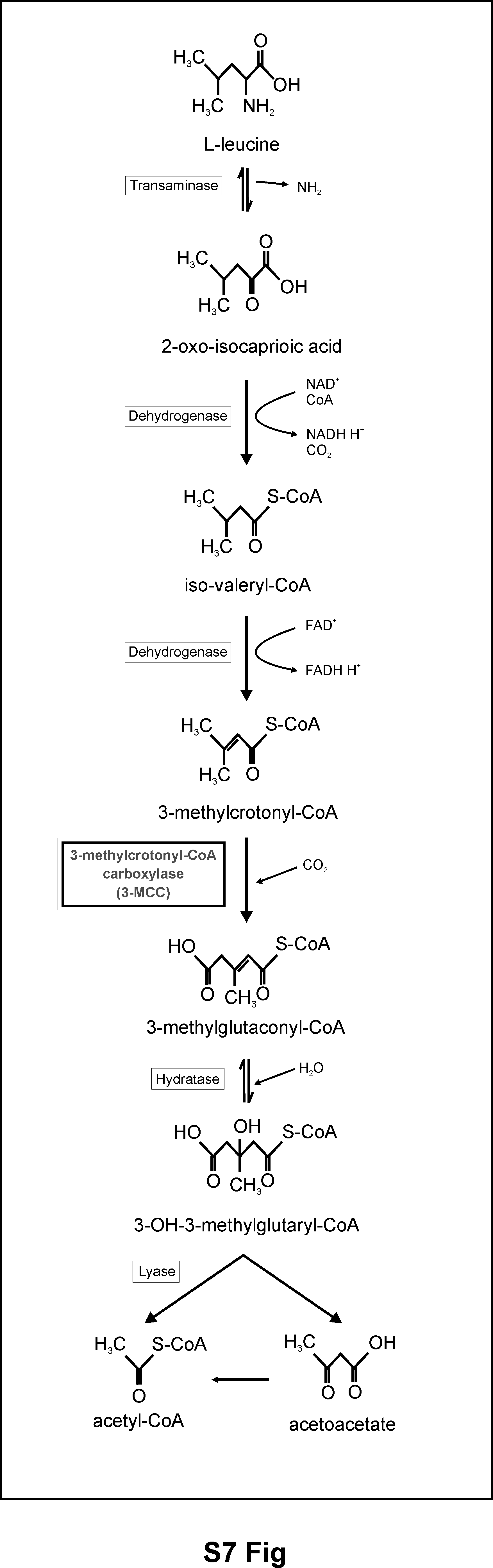

Supplement: S7 Fig — The reactions catalyzed by the enzymes are represented by arrows. The metabolism of the amino acid leucine requires 3-MCC activity (highlighted). The enzymes are: leucine transaminase, 2-ketoisocaproic dehydrogenase, isovaleryl-CoA dehydrogenase, 3-methylcrotonyl-CoA carboxylase, 3-methylglutaconyl-CoA hydratase, 3-hydroxy-3-methylglutaryl-CoA lyase. (TIF) [file pone.0198414.s008.tif]
